# Iron Prevents the Development of Experimental Cerebral Malaria by Attenuating CXCR3-Mediated T Cell Chemotaxis

**DOI:** 10.1371/journal.pone.0118451

**Published:** 2015-03-13

**Authors:** Kristin M. Van Den Ham, Marina Tiemi Shio, Anthony Rainone, Sylvie Fournier, Connie M. Krawczyk, Martin Olivier

**Affiliations:** 1 Department of Microbiology and Immunology, McGill University, Montréal, Québec, Canada; 2 McGill International TB Centre, Research Institute of the McGill University Health Centre, Montréal, Québec, Canada; Institut national de la santé et de la recherche médicale - Institut Cochin, FRANCE

## Abstract

Cerebral malaria is a severe neurological complication of *Plasmodium falciparum* infection. Previous studies have suggested that iron overload can suppress the generation of a cytotoxic immune response; however, the effect of iron on experimental cerebral malaria (ECM) is yet unknown. Here we determined that the incidence of ECM was markedly reduced in mice treated with iron dextran. Protection was concomitant with a significant decrease in the sequestration of CD4^+^ and CD8^+^ T cells within the brain. CD4^+^ T cells demonstrated markedly decreased CXCR3 expression and had reduced IFNγ-responsiveness, as indicated by mitigated expression of IFNγR2 and T-bet. Additional analysis of the splenic cell populations indicated that parenteral iron supplementation was also associated with a decrease in NK cells and increase in regulatory T cells. Altogether, these results suggest that iron is able to inhibit ECM pathology by attenuating the capacity of T cells to migrate to the brain.

## Introduction

The processes contributing to the pathophysiology of experimental cerebral malaria (ECM) are multi-factorial and incompletely understood. Sequestration of immune cells and parasitized red blood cells (pRBCs) in the brain [1–3], activation of the inflammatory response [4,5], and the eventual loss of blood-brain barrier (BBB) integrity [6,7] have been shown to play integral roles in the development of the disease. Numerous studies have demonstrated that CD8^+^ T cells are the principal effector cells involved in the development of pathology [8–10]; depletion of CD8^+^ T cells one day prior to the predicted onset of neurological symptoms results in 100% protection [8]. Moreover, mice deficient in the chemokine receptor CXCR3, or its ligands CXCL9 and CXCL10, show a marked decrease in the incidence of ECM, coincident with reduced trafficking of CD8^+^ T cells to the brain [11,12]. Importantly, IFNγ produced by brain-sequestered CD4^+^ T cells is sufficient to induce the production of CXCL9 and CXCL10, thereby contributing to the accumulation of CXCR3-expressing CD8^+^ T cells within the brain [13,14].

The priming of *Plasmodium berghei* ANKA-specific T cell responses occurs in the spleen [8,15]. MHC I-restricted antigens expressed by blood-stage parasites are captured by dendritic cells (DCs) and cross-presented to naïve CD8^+^ T cells, resulting in cell proliferation and the generation of cytotoxic T lymphocytes [16–18]. The expression of CXCR3 on splenic T cells has been shown to increase during *P. berghei* ANKA infection [19]. This upregulation is thought to be dependent on NK cells, as their depletion results in significantly reduced CXCR3 expression on splenic T cells and decreased accumulation of T cells within the brain [19]. Additionally, the expansion of regulatory T cells (Tregs) *in vivo* has been shown to attenuate the sequestration of conventional T cells within the brain and prevent the development of ECM [20]. Further, adoptive transfer of Tregs has been demonstrated to attenuate CXCR3 expression on CD4^+^ T cells [21].

Previous studies have shown that iron overload can inhibit the production of effector cells [22]. More recently, DCs have been observed to contribute to the generation of the reducing microenvironment required for T cell activation and proliferation [23]. Thus, the resultant increase in oxidative radicals from parenteral iron supplementation may inhibit the development of an efficient immune response [24]. Moreover, Tregs have also been shown to inhibit DC-mediated redox remodelling [25]. However, the ability of iron to potentiate this specific attenuation and the other possible ramifications of augmented iron levels on T cells has not yet been fully elucidated, particularly in the context of a pathogenic disease.

Here we report that parenteral iron supplementation significantly decreased the incidence of ECM, concomitant with a marked reduction in the presence of CD4^+^ and CD8^+^ T cells in the brain. Splenic CD4^+^ and CD8^+^ T cells showed normal activation, but CD4^+^ T cells had decreased CXCR3 expression. Furthermore, CD4^+^ T cells demonstrated evidence of reduced IFNγ-responsiveness, characterized by attenuated IFNγR2 and T-bet expression. Analysis of splenic populations revealed that iron supplementation increased Treg cell numbers and decreased NK cell numbers. Collectively these findings suggest that iron supplementation does not impair T cell activation, but rather alters the ability of T cells to migrate to the brain and cause pathology.

## Results

### Parenteral iron supplementation markedly protects mice from ECM

**Fig 1:**
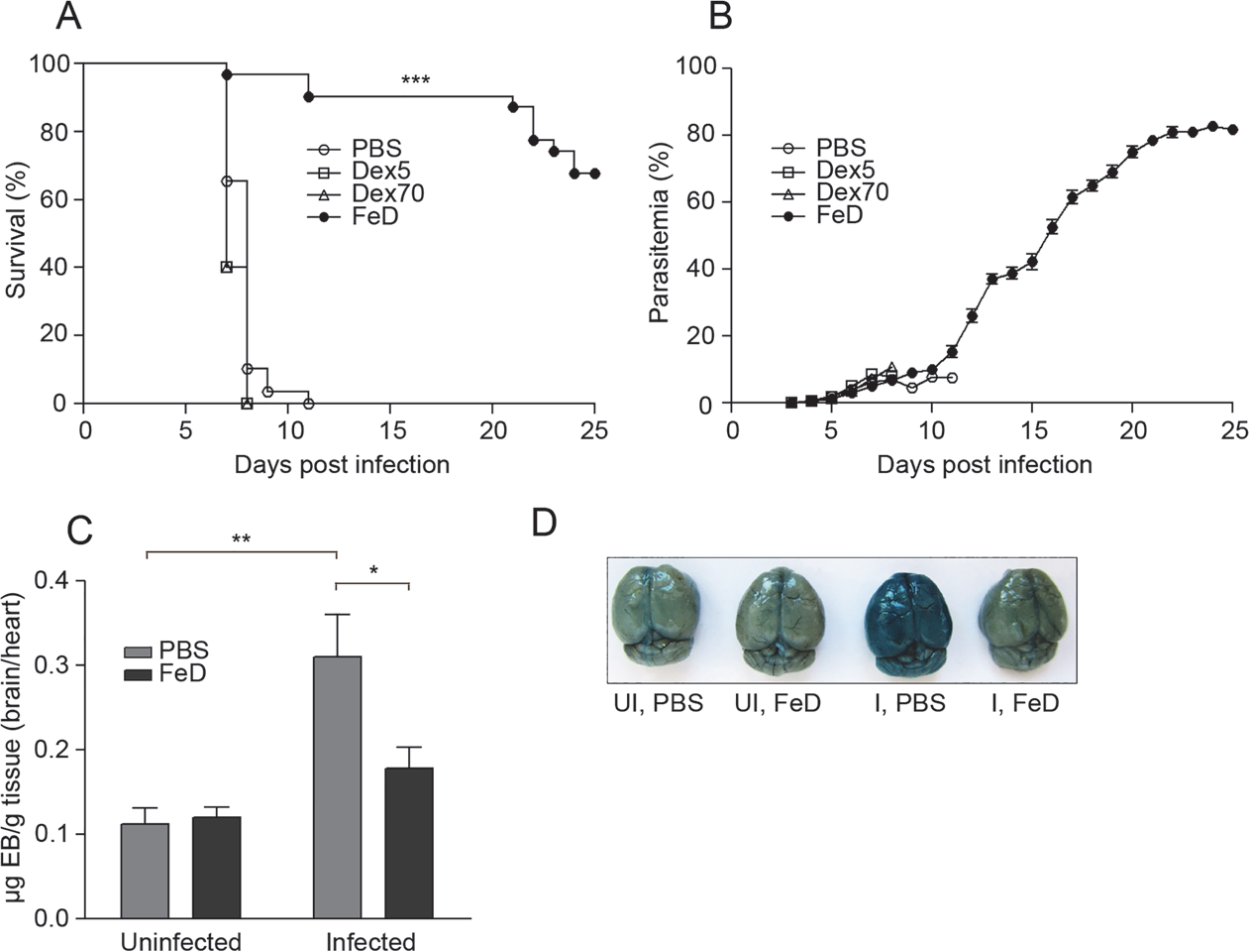
Iron Dextran Prevents the Development of ECM. Survival (**a**) and mean parasitemia (**b**) of infected mice treated with iron dextran, dextran M_w_ 5kDa and dextran M_w_ 70kDa. PBS-treated mice were used as a control. BBB disruption was assessed using Evans blue (EB). EB quantification (**c**) is shown as mean μg of EB per g of brain tissue normalized to mean μg of EB per g of heart tissue. Representative picture of EB-stained brains (**d**). For survival: *n* = 29 for control mice, *n* = 31 for FeD mice and *n* = 5 for Dex5 and Dex70 mice. The average of four individual experiments is shown for the control and FeD mice. For parasitemia: *n* = 20 for control and FeD mice and *n* = 5 for Dex5 and Dex70. The average of three individual experiments is shown for the control and FeD mice. For EB: *n* = 5 for control, uninfected and infected mice, and *n* = 6 for iron dextran-treated, uninfected and infected mice. PBS = control, Dex5 = dextran M_w_ 5kDa, Dex70 = dextran M_w_ 70kDa, FeD = iron dextran, UI = uninfected, I = infected. Statistically significant differences, shown by asterisks (* *P* < 0.05, ** *P* < 0.01, and *** *P* < 0.001), were determined by log-rank test (survival) and unpaired Student’s t-test (BBB disruption).

To determine the effect of parenteral iron supplementation on the pathology of ECM, we infected iron dextran-treated (FeD) mice with *P. berghei* ANKA. C57BL/6 mice infected with this parasite develop clinical symptoms of ECM (i.e., hemi- or paraplegia, convulsions and coma) and succumb to the disease within 6 to 9 d post-infection [26,27]. Dextran controls (with M_w_ = 5,000 kDa and 70,000 kDa) were included to establish if the dextran component itself was augmentative. PBS-treated (control) and dextran-treated mice developed ECM between days 7 and 11 post-infection, and their mortality was 100% (**Fig. 1A**). In contrast, FeD mice were markedly protected, with a mortality of only 0–20% (**Fig. 1A**). Additionally, further experiments revealed that iron supplementation provided a slight, but significant, protective effect when started up to 4 days post-infection (**Figure A in S1 File**). The level of parasitemia for control, dextran-treated and FeD mice was found to be similar through day 11 post-infection, by which time all control and dextran-treated mice had succumbed to ECM (**Fig. 1B**). This result suggests that the protective effect of iron dextran does not rely on the inhibition of parasitemia. FeD mice that did not develop ECM had increasing levels of parasitemia and either died due to the development of hyperparasitemia (pRBC > 80%) or were sacrificed on day 25 post-infection (**Fig. 1B**). Since the mice treated with the dextran controls had the same clinical phenotype as the control mice, the iron-mediated protection was further investigated using only the control mice. ECM incidence was confirmed by analyzing the integrity of the BBB, which is a hallmark of ECM pathology [6]. The uptake of Evans blue (EB) into the brain parenchyma, which is indicative of BBB disruption, was evident in the infected control mice and was significantly reduced in the FeD mice (**Fig. 1C, D**), indicating that iron supplementation prevented the loss of BBB integrity during ECM.

### Organ sequestration of pRBC is decreased in FeD mice

**Fig 2:**
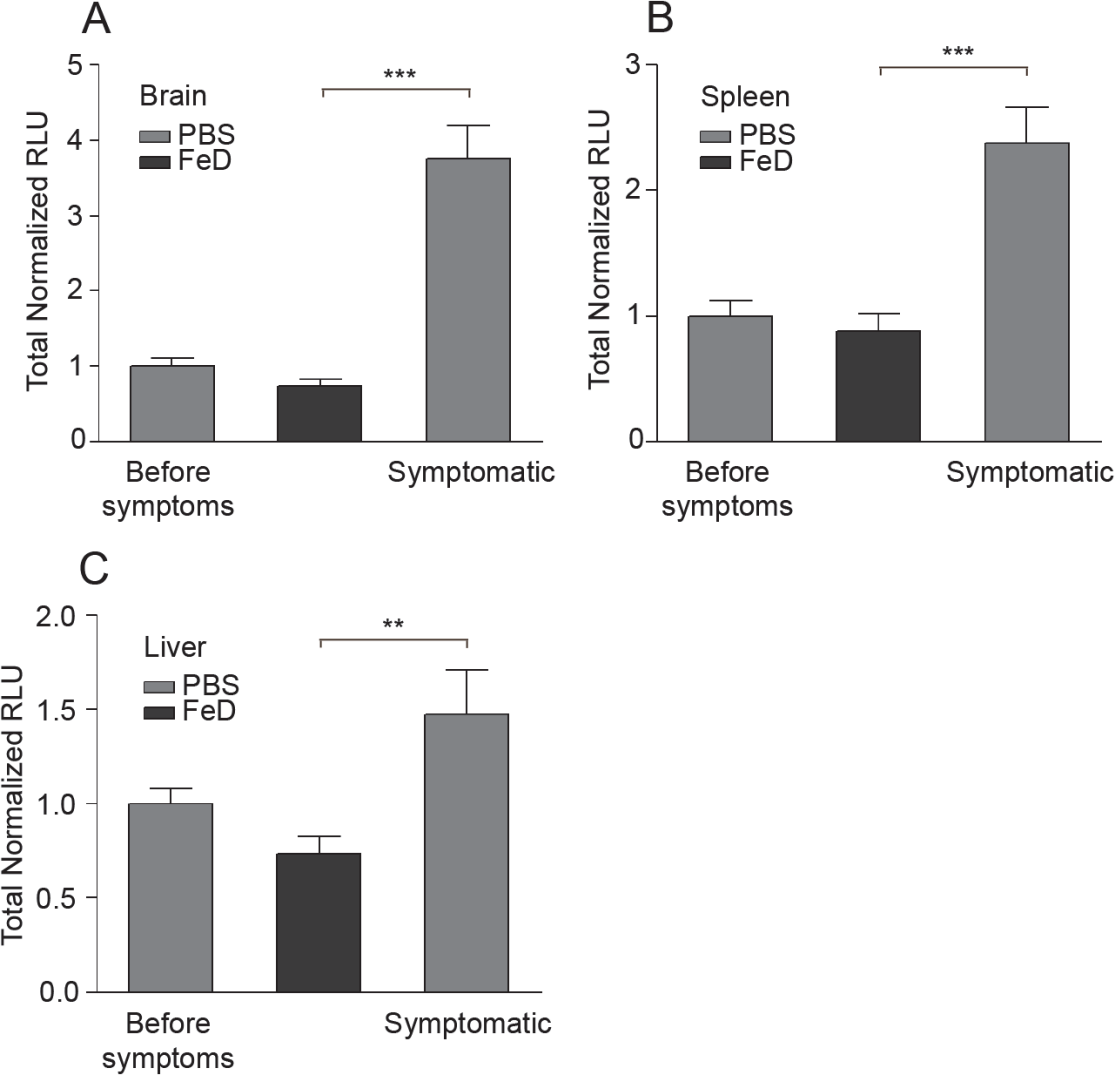
Tissue parasite sequestration is inhibited by parenteral iron supplementation. Parasite levels in the brain (**a**), spleen (**b**) and liver (**c**) on day 7 post-infection. Luciferase activity is shown as the total RLU per organ normalized to the total RLU in the control mice before symptoms. *n* = 8 for the control mice before symptoms, *n* = 8 for the control, symptomatic mice and *n* = 12 for the FeD mice. FeD = iron dextran, PBS = control. Statistically significant differences, shown by asterisks (** *P* < 0.01 and *** *P* < 0.001), were determined by unpaired Student’s t-test.

Recent studies have shown that a rapid increase in tissue parasite burden, independent of parasitemia, is associated with the induction of clinical ECM [28–32]. Parasite burden in the brain, spleen and liver were measured on day 7 post-infection using luciferase activity. Levels of parasitemia assessed using luciferase activity were similar to parasitemia as measured by counting blood smears, indicating that the two methods are comparable (**Figure B.A in S1 File**). Although a significant difference in the parasitemia between the control and FeD mice was not observed before the development of symptoms in the control mice, a marked increase in tissue parasite burden was observed in the control mice upon the development of clinical symptoms (**Fig. 2A-C**). An abrupt increase in the tissue parasite levels was not observed in FeD mice, as they did not become symptomatic. Since the tissue parasite burden in the control mice only increased upon the development of symptoms, the surge in parasites is likely associated with the onset of ECM pathology, which is in agreement with previous studies [28–32]. In the control mice the parasite burden increased to a greater extent in the brain compared to the spleen and the liver. However, parasite sequestration in the infected FeD mice and the infected control mice before the development of symptoms (as measured by the ratio of the relative luminescence units (RLU) of the infected FeD mice or the infected control before the development of symptoms to the RLU of the uninfected mice) was much more extensive in the spleen (2250 fold increase) and liver (40 fold increase) than in the brain (3 fold increase) (**Figure B.B-D in S1 File**). This large variance may account for the apparent lack of parasite sequestration detected in the brain of infected mice in some earlier studies [33].

### FeD mice have an increased systemic inflammatory response

**Fig 3:**
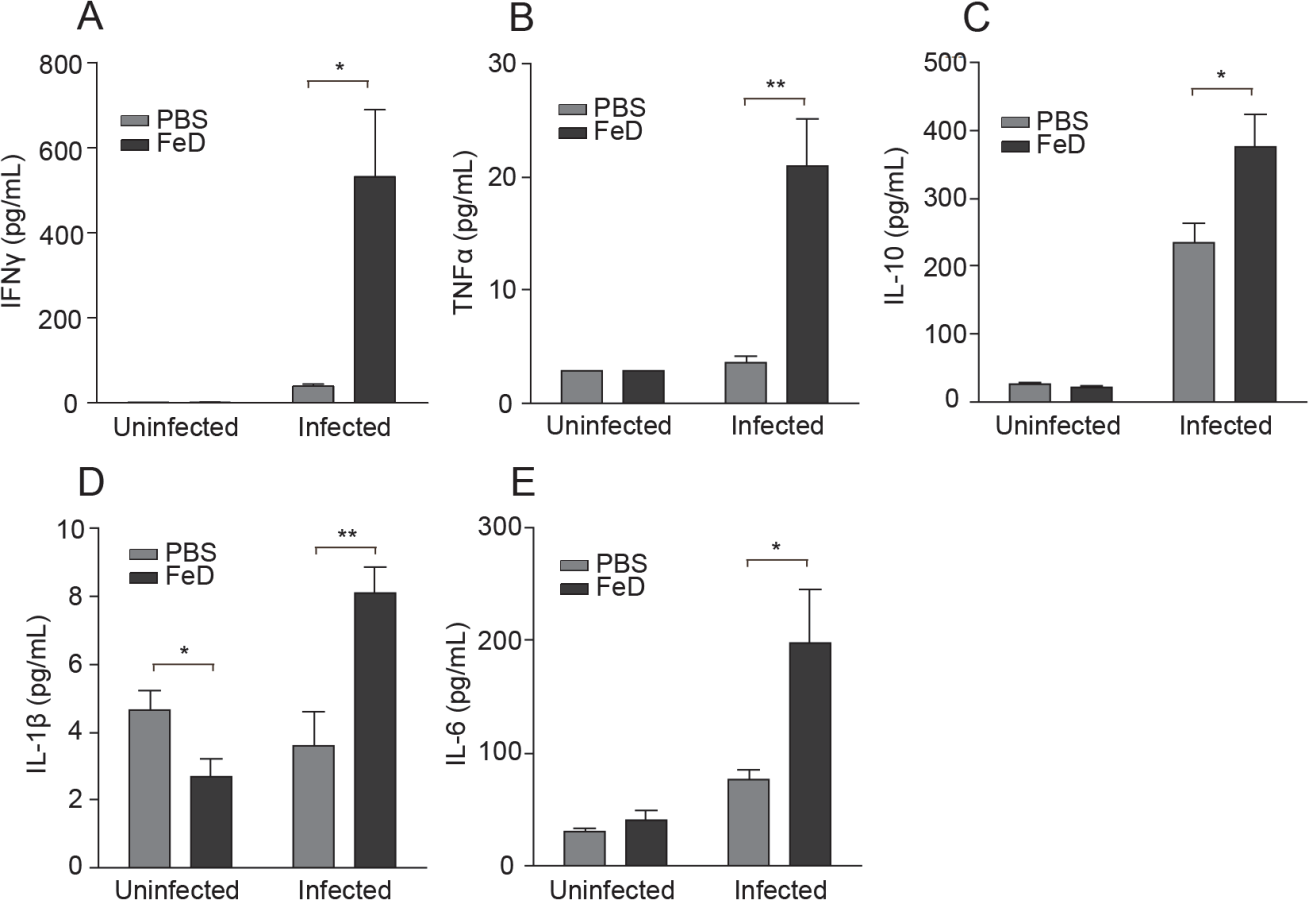
Systemic Inflammation is Augmented in FeD Mice. Concentration of IFNγ (**a**), TNFα (**b**), IL-10 (**c**), IL-1β (**d**) and IL-6 (**e**) in the serum on day 7 post-infection. *n* = 5 for the control, uninfected group mice and *n* = 6 for all other groups. Levels of TNFα are below the limit of detection in the uninfected groups. FeD = iron dextran, PBS = control. Statistically significant differences, shown by asterisks (* *P* < 0.05 and ** *P* < 0.01), were determined by unpaired Student’s t-test.

The activation of the inflammatory response by parasite antigens and immune cell adhesion has been shown to contribute to ECM pathology. Therefore, to discern the effect of parenteral iron supplementation on the systemic immune response, the concentrations of several cytokines in the serum were measured on day 7 post-infection. Overall, our data suggests that iron supplementation increased the proinflammatory response at late time points during *P. berghei* ANKA infection. Importantly, administration of iron dextran without infection did not appear to have a significant effect on most of the inflammatory mediators analyzed (**Fig. 3A-E**). Surprisingly, IFNγ, which has been established to be integral to the development of ECM pathology [13,14,28,29,34], was observed to be significantly increased in the FeD mice compared to the control mice (**Fig. 3A**). The FeD mice also had increased concentrations of TNFα (**Fig. 3B**) and IL-10 (**Fig. 3C**) after infection. TNFα and IL-10 have been shown to be associated with decreased tissue parasite accumulation during ECM [29,35], and this may account for the observed reduction in parasite burden in the FeD mice (**Fig. 2A-C**). Furthermore, the FeD mice exhibited an increased serum concentration of IL-1β (**Fig. 3D**) and IL-6 (**Fig. 3E**), indicating a strong inflammatory response, but neither cytokine has been shown to play a role in the development of ECM [36,37]. The levels of cytokines in the serum were also measured over the course of the infection. The serum concentration of IFNγ, TNFα, IL-10 and IL-6 only started to increase substantially on day 6 post-infection, and no apparent difference was observed in the serum levels of IL-1β (**Figure C in S1 File**). Interestingly, in contrast to the results obtained for the serum, the expression of inflammatory and immune response-related genes in the brain, spleen and liver of the FeD mice was predominately unchanged or reduced compared to the control mice (**Table A-C in S1 File**).

### Expression of genes involved in T cell chemotaxis is attenuated in FeD mice

**Fig 4:**
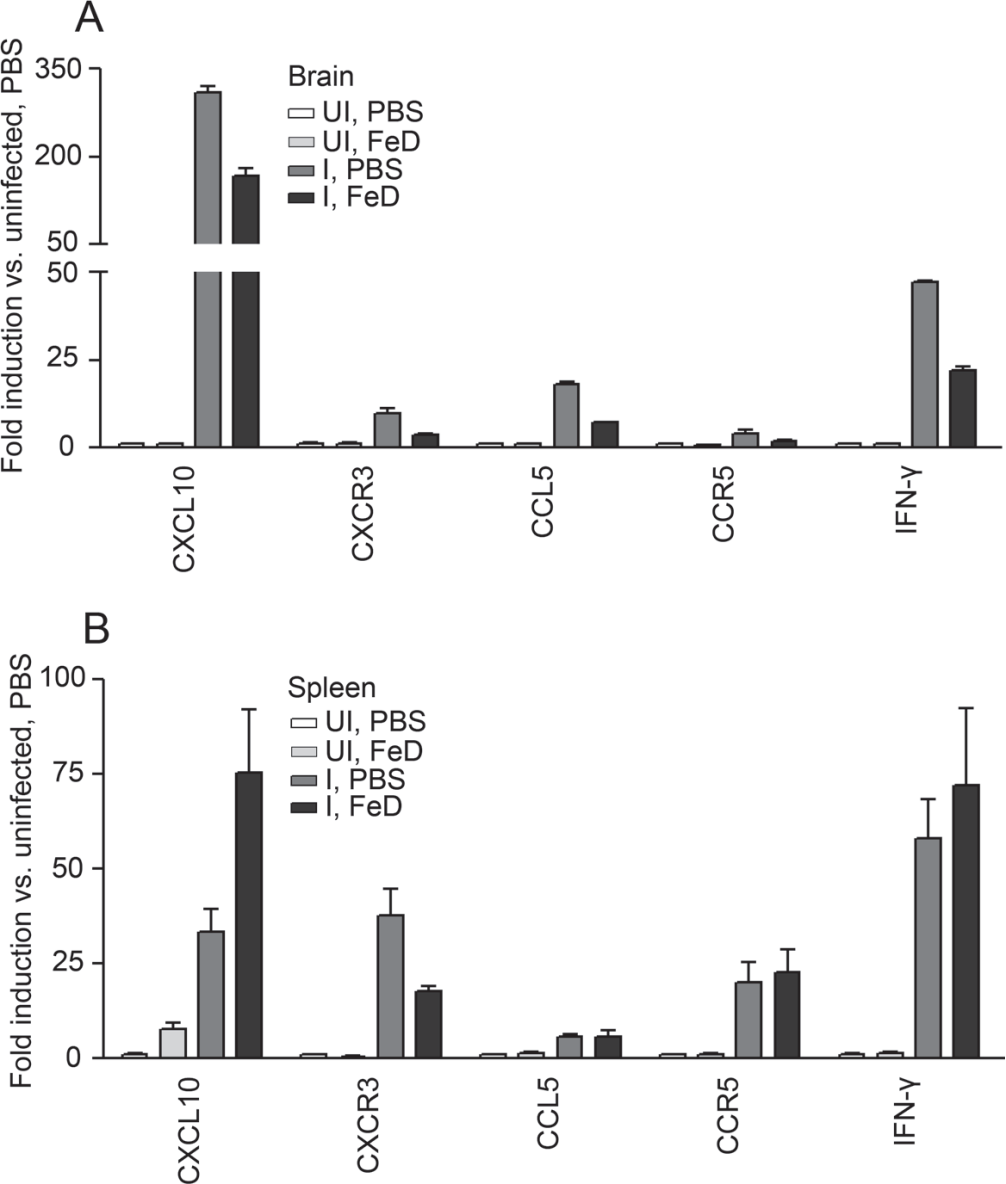
The Expression of Genes Involved in T Cell Chemotaxis are Attenuated by Parenteral Iron Supplementation. The expression of genes involved in T cell chemotaxis is shown for the brain (**a**) and the spleen (**b**) on day 7 post-infection. mRNA levels were normalized to *Gusb*. 2 samples pooled from 6 mice (3 mice per sample) were used for each group. UI = uninfected, I = infected, FeD = iron dextran, PBS = control.

The expression of genes involved in the immune response was further profiled in the brain and the spleen on day 7 post-infection to develop a more comprehensive understanding of how parenteral iron supplementation may have been facilitating protection during ECM. Numerous studies have demonstrated that CD8^+^ T cell accumulation within the brain is necessary for the clinical onset of ECM [8–14,19,28–32,38]. In the brain, it was observed that multiple genes involved in T cell trafficking were downregulated (**Fig. 4A**). The mRNA expression of chemokines (CXCL10 and CCL5) and chemokine receptors (CXCR3 and CCR5) that have previously been determined to play a role in T cell trafficking and ECM pathogenesis were decreased [11,12,39]. IFNγ production by CD4^+^ T cells has been shown to contribute to the accumulation of CD8^+^ T cells in the brain by inducing the expression of chemokines [13,14]. Expression of IFNγ in the brain was also observed to be reduced in the FeD mice. Since T cell priming during ECM occurs in the spleen [8,15], we also analyzed the expression of genes involved in T cell trafficking in this organ (**Fig. 4B**). In the spleen, a large increase in the expression of CXCL10 and a decrease in the expression of CXCR3 were detected in FeD mice; whereas the expression of CCL5 and CCR5 were unchanged by iron supplementation. Additionally, the expression of IFNγ in the spleen was unchanged. This data suggests that CD4^+^ and CD8^+^ T cells in the FeD mice have an impaired ability to traffick to the brain during *P. berghei* ANKA infection.

### Sequestration of CD4^+^ and CD8^+^ T cells in the brain is greatly reduced in FeD mice

**Fig 5:**
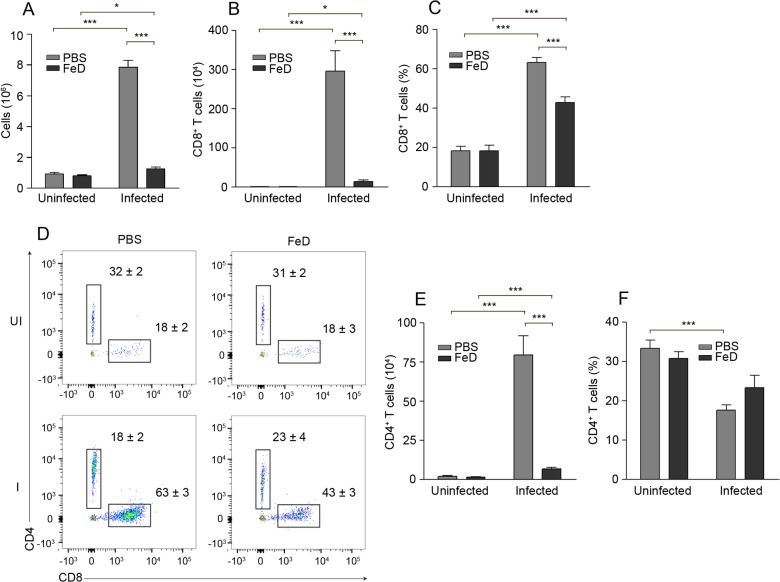
Sequestration of CD4^+^ and CD8^+^ T Cells in the Brain is Reduced in FeD Mice. The total number of cells recovered after isolation (**a**), the total number (**b**) and percentage (**c**) of CD8^+^ T cells after gating on infiltrating leukocytes (CD45^+^CD11b^lo-hi^), representative flow cytometric dot plots of CD4^+^ and CD8^+^ T cells after gating on infiltrating leukocytes (**d**), and the total number (**e**) and percentage (**f**) of CD4^+^ T cells after gating on infiltrating leukocytes, on day 7 post-infection. The numbers shown on the dot plots indicate the mean percentage of cells inside the gate ± S.E.M. *n* = 5 mice were used for each group. UI = uninfected, I = infected, FeD = iron dextran, PBS = control. Statistically significant differences, shown by asterisks (* *P* < 0.05 and *** *P* < 0.001), were determined by unpaired Student’s t-test.

The sequestration of CD4^+^ and CD8^+^ T cells to the brain was examined on day 7 post-infection to determine if the decreased expression of trafficking-associated genes correlated with a reduction in the chemotaxis of T cells to the brain. Infiltration of cells into the brain, as represented by the number of cells recovered after cell isolation, was significantly decreased in the FeD mice compared to the control mice after infection (**Fig. 5A**). A slight increase in cell accumulation in the infected FeD mice compared to the uninfected FeD mice was measured, indicating that immune cell recruitment was not completely abrogated by parenteral iron supplementation. Furthermore, the infiltration of cells was not changed between the uninfected control and the uninfected FeD mice, suggesting that iron-mediated attenuation of immune cell sequestration only occurs after infection. There was a marked decrease in the number of accumulated CD8^+^ T cells in the FeD mice after infection, and no change between the control and FeD mice without infection (**Fig. 5B**). Moreover, the number of CD8^+^ T cells was increased in the infected FeD mice compared to the uninfected FeD mice. Similar results were obtained for the percentage of CD8^+^ T cells (**Fig. 5C,D**). A decrease in the total number of CD4^+^ T cells sequestered in the brain of the infected FeD mice compared to the infected control mice was observed (**Fig. 5E**). No difference in the accumulation of CD4^+^ T cells was detected between the control and FeD mice without infection and a minor increase in the number of CD4^+^ T cells was measured in the infected FeD mice compared to the uninfected FeD mice. Iron supplementation did not change the percentage of CD4^+^ T cells, but the percentage decreased during infection in the control mice (**Fig. 5D,F**).

### CXCR3 expression on CD4^+^ T cells in the spleen is significantly decreased in FeD mice

**Fig 6:**
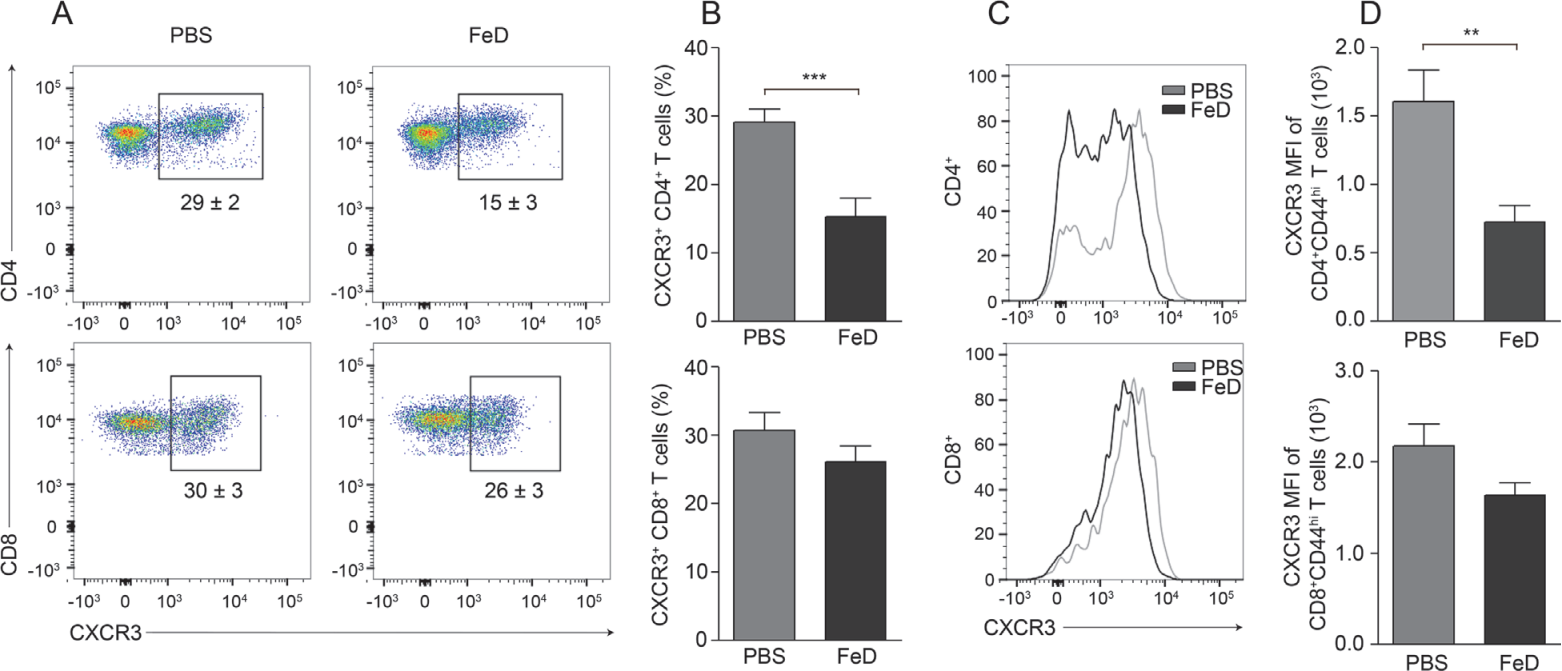
The Expression of CXCR3 on Splenic CD4^+^ T Cells is Decreased by Parenteral Iron Supplementation. Representative flow cytometric dot plots of CXCR3^+^ CD4^+^ and CD8^+^ T cells (**a**) and the percentage of CXCR3^+^ cells after gating on CD4^+^ or CD8^+^ T cells (**b**). Representative flow cytometric histograms of CXCR3 (**c**) and the MFI of CXCR3 (**d**) after gating on CD4^+^CD44^hi^ or CD8^+^CD44^hi^ T cells. All experiments were performed on day 7 post-infection. The numbers shown on the dot plots indicate the mean percentage of cells inside the gate ± S.E.M. *n* = 13 for all groups. The average of two individual experiments is shown. FeD = iron dextran, PBS = control. Statistically significant differences, shown by asterisks (** *P* < 0.01 and *** *P* < 0.001), were determined by unpaired Student’s t-test.

The above results indicated that parenteral iron supplementation prevented ECM pathology by reducing the sequestration of both CD4^+^ and CD8^+^ T cells within the brain. We hypothesized that the attenuated accumulation was due to either a deficiency in activation and/or expansion or to a defect in the chemotaxis of the T cells. The expansion of splenic CD4^+^ and CD8^+^ T cells was very similar between the control and FeD mice; only a minor delay in the proliferation of CD4^+^ T cells was observed (**Figure D.A,B in S1 File**). Additionally, the percentage of activated CD4^+^ and CD8^+^ T cells in the spleen was slightly decreased in the FeD mice, except for the percentage of CD25^+^CD62L^lo^ CD4^+^ T cells, which was unchanged (**Figure E.A,B in S1 File**). Splenic conventional DCs (cDCs) were also analyzed, since this subset of DCs is thought to be responsible for priming the T cell response during ECM [16–18]. No difference in the percentage of cDCs or the mean fluorescence intensity (MFI) of CXCL10, CD40 or MHCII was observed in the FeD mice compared to the control mice (**Figure F.B in S1 File**). Both CXCR3 and CCR5 have been implicated to play important roles in T cell migration during ECM, but previous studies have shown that a greater percentage of brain-infiltrating T cells express CXCR3 compared to CCR5 [19]. Moreover, CXCR3 mRNA expression in the spleen was decreased in the FeD mice, whereas CCR5 expression was unchanged (**Fig. 4B**). Therefore, the expression of CXCR3 on splenic CD4^+^ and CD8^+^ T cells was examined on day 7 post-infection to determine if iron supplementation was attenuating T cell chemotaxis. The percentage of CD4^+^ T cells expressing CXCR3 was markedly decreased in the iron supplemented mice (**Fig. 6A,B**). Furthermore, the MFI of CXCR3 on CD4^+^CD44^hi^ T cells in the FeD mice was similarly reduced (**Fig. 6C,D**). However, the percentage of CXCR3^+^ CD8^+^ T cells was unchanged (**Fig. 6A,B**), as was the MFI of CXCR3 on CD8^+^CD44^hi^ T cells (**Fig. 6C,D**). The expression of CXCR3 on CD8^+^ T cells in the FeD mice trended toward a slight decrease, and a significant reduction was measured in some of the individual experiments, but overall, a significant difference was not observed. The attenuated expression of CXCR3 on CD4^+^ T cells suggests that iron supplementation inhibits T cell sequestration within the brain by directly impairing the chemotactic capacity of only CD4^+^ T cells, and that the chemotaxis of CD8^+^ T cells to the brain is indirectly attenuated by the consequent decrease in the induction of chemokines by CD4^+^ T cells.

### IFNγR2 and T-bet expression in splenic T cells in FeD mice is attenuated during ECM

cDCs and CD4^+^ T cells in the spleen were analyzed on day 3 post-infection to determine if the cause of the attenuated expression of CXCR3 on CD4^+^ T cells was due to an early defect in activation or to impaired differentiation. No difference in the percentage of cDCs or the MFI of CXCL10 was observed, but a modest increase in the MFI of CD40 and MHCII was detected in the FeD mice (**Figure F.A in S1 File**). Interestingly, on day 3 post-infection, a greater percentage of splenic CD4^+^ T cells in the FeD mice had an activated phenotype and expressed CXCR3 (**Figure G.A-D in S1 File**). This result is in contrast to the status of CD4^+^ T cells on day 7 post-infection, at which time point CD4^+^ T cells from the FeD mice had a slight decrease in activation (**Figure E.A,B in S1 File**) and a marked decrease in CXCR3 expression (**Fig. 6A-D**). IFNγ signalling through the IFNγR (IFNγR1/IFNγR2) induces T-bet, which subsequently transactivates both IFNγ and CXCR3 [40]. The limiting factor in IFNγ-responsiveness is the expression of IFNγR2, which has been shown to be downregulated by both IFNγ [41] and iron [42]. Consequently, the expression of IFNγR2 and T-bet was measured to develop a better understanding of CXCR3 induction in splenic CD4^+^ T cells.

**Fig 7:**
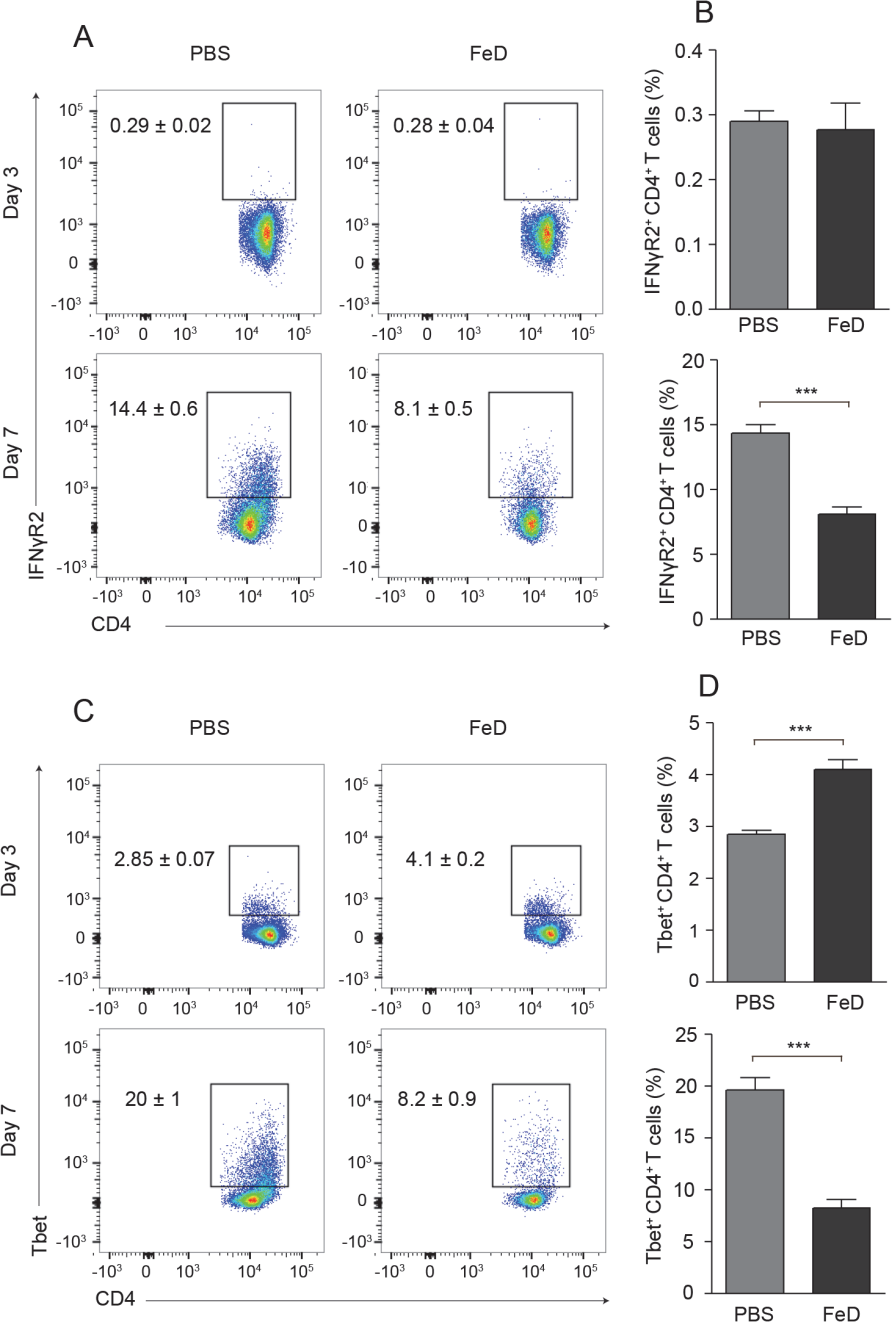
Iron Dextran Mitigates the Upregulation of IFNγR2 and T-bet on CD4^+^ T Cells in the Spleen. Representative flow cytometric dot plots for IFNγR2^+^ CD4^+^ T cells (**a**) and the percentage of IFNγR2^+^ cells (**b**) after gating on CD4^+^ T cells on day 3 and day 7 post-infection. Representative flow cytometric dot plots for T-bet^+^ CD4^+^ T cells (**c**) and the percentage of T-bet^+^ cells (**d**) after gating on CD4^+^ T cells on day 3 and 7 post-infection. The numbers shown on the dot plots indicate the mean percentage of cells inside the gate ± S.E.M. On day 3 post-infection, *n* = 6 for control mice and *n* = 5 for FeD mice. On day 7 post-infection, *n* = 6 for control mice and *n* = 6 for FeD mice. FeD = iron dextran, PBS = control. Statistically significant differences, shown by asterisks (*** *P* < 0.001), were determined by unpaired Student’s t-test.

On day 3 post-infection, IFNγR2 was expressed on a low percentage of CD4^+^ T cells and the percentage of IFNγR2^+^ CD4^+^ T cells was not significantly different in the FeD mice compared to the control mice (**Fig. 7A,B**). The percentage of T-bet^+^ CD4^+^ T cells on day 3 post-infection was increased in the FeD mice (**Fig. 7C,D**), concurring with the increased percentage of CXCR3^+^ CD4^+^ T cells observed on this day (**Figure G.C,D in S1 File**). The percentages of IFNγR2- and T-bet-expressing CD4^+^ T cells were increased in both groups on day 7 post-infection compared to day 3 post-infection; however, the percentages of IFNγR2^+^ and T-bet^+^ CD4^+^ T cells were markedly reduced in the FeD mice on day 7 post-infection (**Fig. 7A-D**). This result agrees with the decreased expression of CXCR3 measured on CD4^+^ T cells in the FeD mice on day 7 post-infection (**Fig. 6A-D**). No differences were observed in the expression of IFNγR2 or T-bet on CD8^+^ T cells on day 3 post-infection, but the expression of both IFNγR2 and T-bet were significantly decreased in the FeD mice on day 7 post-infection (**Figure H.A-D in S1 File**). However, this did not culminate in a decrease in CXCR3 expression (**Fig. 6A-D**). IFNγ stimulation induces T-bet through the activation of STAT1 [40]; therefore, the phosphorylation of STAT1 was examined to verify that iron supplementation was inhibiting IFNγ signalling in CD4^+^ T cells. The phosphorylation of STAT1 was notably reduced in CD4^+^ T cells in the FeD mice on day 7 post-infection (**Figure I in S1 File**). Thus, these results suggest that the attenuated expression of CXCR3 on splenic CD4^+^ T cells is dependent on the iron-mediated decrease in the IFNγ-responsiveness of CD4^+^ T cells.

### FeD mice have a reduction in NK cells and an augmentation of Tregs in the spleen

**Fig 8:**
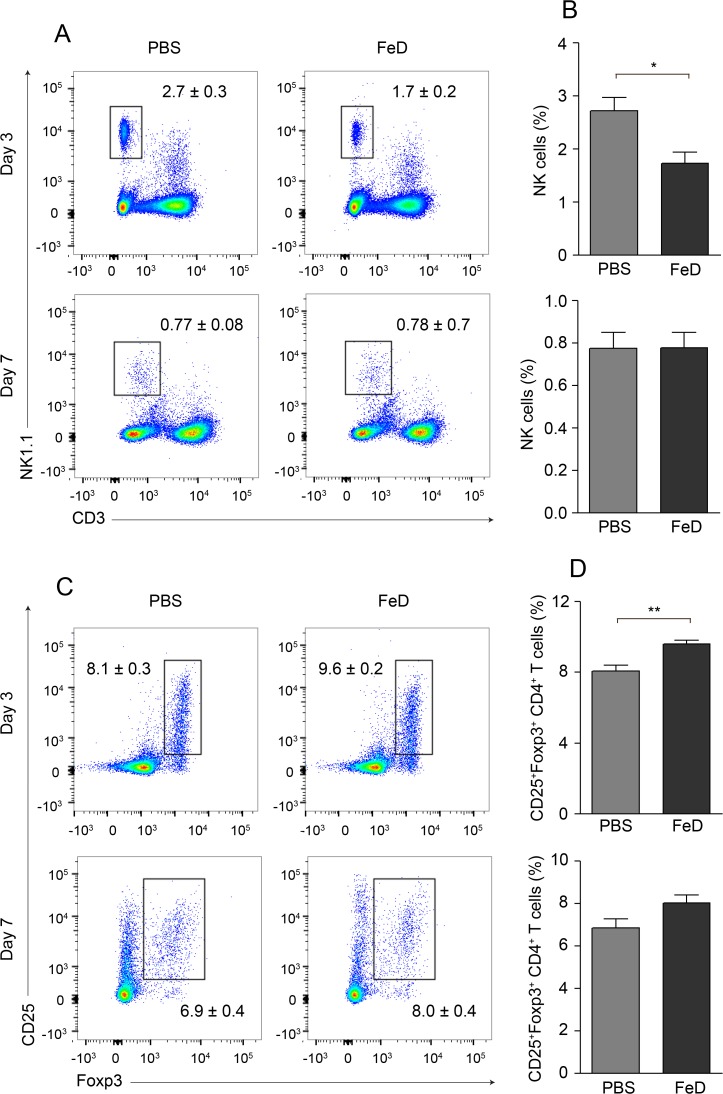
FeD Mice have Modulated Frequencies of Splenic NK Cells and Tregs Early During the Infection. Representative flow cytometric dot plots of NK cells (**a**) and the percentage of NK cells (**b**) on day 3 and day 7 post-infection. Representative flow cytometric dot plots of Tregs (**c**) and the percentage of Tregs after gating on CD4^+^ T cells (**d**) on day 3 and day 7 post-infection. The numbers shown on the dot plots indicate the mean percentage of cells inside the gate ± S.E.M. On day 3 post-infection, *n* = 6 for control mice and *n* = 5 for FeD mice, except for Tregs, where *n* = 5 for the control mice. On day 7 post-infection, *n* = 6 for control mice and *n* = 6 for FeD mice. FeD = iron dextran, PBS = control. Statistically significant differences, shown by asterisks (* *P* < 0.05 ** *P* < 0.01), were determined by unpaired Student’s t-test.

Finally, we were interested in determining if iron supplementation affected other factors that might augment protection. Both NK cells and Tregs have been shown to influence the development of ECM. Depletion of NK cells using an anti-asialo GM1 antibody has been shown to inhibit T cell chemotaxis to the brain by attenuating CXCR3 expression on splenic T cells [19]. Additionally, the *in vivo* expansion of Tregs was observed to prevent conventional T cell accumulation in the brain during ECM through a CTLA-4-dependent mechanism [20]. This study did not examine the expression of CXCR3 on conventional T cells; however, adoptive transfer of Tregs has previously been demonstrated to mitigate CXCR3 expression on CD4^+^ T cells [21]. Therefore, the frequencies of splenic NK cells and Tregs were measured to determine if iron conferred protection by modulating the percentages of these cell types. On day 3 post-infection, the percentage of splenic NK cells was markedly decreased in the FeD mice (**Fig. 8A,B**). The percentage of NK cells decreased in both groups from day 3 post-infection to day 7 post-infection and the percentage of NK cells in the FeD mice was the same as the control mice on day 7 post-infection (**Fig. 8A,B**). The percentage of Tregs was increased in the FeD mice on day 3 post-infection (**Fig. 8C,D**). This trend was also observed on day 7 post-infection; however, the difference was no longer significant (**Fig. 8C,D**). Therefore, it appears that the modulated frequencies of NK cells and Tregs are associated with decreased ECM pathology in the FeD mice.

## Discussion

Iron status has been shown to affect the pathogenicity of numerous infections, including malaria, with iron supplementation generally being associated with increased susceptibility [43]. However, populations that have the greatest risk for developing malaria are also those that have an increased frequency of iron deficiency anemia. Moreover, malaria infection itself has been shown to contribute to iron deficiency by modulating the distribution and utilization of iron [44]. Additionally, the complex relationship between host iron status and malaria infection also includes the proposed utilization of iron chelation as an ancillary therapy. Iron deficiency has been previously observed to be associated with decreased risk of developing parasitemia and severe malaria [45]. Anemic hosts have decreased reticulocyte production (the preferred host cell of *P. vivax*) [46] and phagocytize pRBCs more efficiently [47].

However, inhibition of malaria by iron chelators is independent of host iron status, and instead relies on intracellular chelation of the labile iron pool within the pRBCs (iron (III) chelators; *e.g.*, desferrioxamine B) or the formation of toxic complexes with iron (iron (II) chelators; *e.g.*, 2’,2’-bipyridyl) [48–50]. Furthermore, a review of the clinical trials using iron chelators found insufficient evidence to support the use of iron chelation as an adjunctive therapy for malaria [51]. Nevertheless, an improved understanding of how *Plasmodium* parasites acquire iron and how host iron status affects the immune response during malaria infection would greatly aid iron supplementation guidelines in malaria endemic areas. Thus, we sought to determine the impact of parenteral iron supplementation on the development of severe malaria

Mice treated with iron dextran had a markedly reduced incidence of cerebral malaria. The iron-mediated protection did not appear to be through an anti-malarial mechanism, as the parasitemia was not significantly different in the FeD mice compared to the control mice. However, the FeD mice had a reduced tissue parasite burden compared to the control mice that developed symptoms. Previous studies have shown that the accumulation of parasites in the brain and spleen is dependent on the sequestration of CD8^+^ T cells and the production of IFNγ and other inflammatory mediators [28,29]. Therefore, the reduced tissue parasite burden observed in the FeD mice suggests that iron administration provided protection against ECM by attenuating CD8^+^ T cell sequestration and/or signalling by proinflammatory cytokines.

The sequestration of CD8^+^ T cells in the brain has been established to be essential to the development of ECM pathology, by both augmenting parasite accumulation [28–32] and by directly damaging the BBB [9,31,38]. The total number of both CD4^+^ and CD8^+^ T cells sequestered in the brain was markedly decreased in the FeD mice, suggesting that iron supplementation was attenuating the expansion, activation or chemotactic capacity of the splenic T cells. Only a minor delay in proliferation of CD4^+^ T cells and a slight reduction in the activation of CD4^+^ and CD8^+^ T cells were observed in the FeD mice. However, the percentage of CXCR3^+^ CD4^+^ T cells was greatly reduced in the FeD mice, as was the MFI of CXCR3 on CD4^+^CD44^+^ T cells. Contrastingly, the expression of CXCR3 on CD8^+^ T cells was unchanged in the FeD mice compared to the control mice. Earlier studies demonstrated that IFNγ production by brain-sequestered CD4^+^ T cells promotes T cell chemotaxis to the brain by inducing the expression of CXCL9 and CXCL10 [13,14]. Therefore, the reduced chemotactic capacity of splenic CD4^+^ T cells is likely responsible for the decreased expression of CXCL10 in the brain and the consequent reduction in the brain sequestration of CXCR3-expressing CD8^+^ T cells.

Moreover, IFNγ production has been established to be essential to the development of ECM pathology, as both IFNγ^-/-^ and IFNγR^-/-^ mice are completely protected from developing cerebral malaria [13,14,28,29,34]. In an apparent contradiction to previous studies, the FeD mice had significantly higher concentrations of IFNγ in the blood than the control mice. However, IFNγR^-/-^ mice also have increased concentrations of IFNγ in the blood compared to wild-type mice [34]. It was proposed that the concentrations were higher in the knock-out mice because the IFNγ released into the blood could not bind to the receptor, and therefore was not as rapidly eliminated [34]. The expression of IFNγR2 on splenic CD4^+^ T cells was reduced on day 7 post-infection in the FeD mice. Previous studies have shown that the expression of the IFNγR2 chain can be downregulated by both iron [42] and IFNγ [41]. Thus, the decreased expression of IFNγR2 in the FeD mice was likely caused by the augmented level of iron and potentially reinforced by the increasing levels of IFNγ. Furthermore, IFNγ signalling has been demonstrated to be necessary for the induction of CXCR3 on CD4^+^ T cells, but not on CD8^+^ T cells [52,53]. We observed that parenteral iron supplementation attenuated the expression of IFNγR2 and T-bet on splenic CD4^+^ and CD8^+^ T cells, mitigating the IFNγ signalling capacity of these cells. The decrease in the IFNγ-responsiveness of the T cells may account for the expression of CXCR3 being significantly decreased on CD4^+^ T cells and relatively unaffected on CD8^+^ T cells.

Finally, iron administration resulted in a decrease in NK cells and increase in Tregs in the spleen. The depletion of NK cells and the *in vivo* expansion of Tregs have both been shown to protect mice from developing ECM by attenuating T cell sequestration within the brain [19,20]. The modulated frequencies of NK cells and Tregs in the FeD mice correlated with the decreased expression of CXCR3 on the splenic CD4^+^ T cells; however, these cell types could potentially be acting through mechanisms other than chemotaxis inhibition. Additionally, as dextran was not used as a control throughout the study, the ability to identify the changes induced by dextran itself is limited; however, it is unlikely that dextran plays a major role in the observed alterations. Previous studies have shown that iron treatment causes T cells to become refractory to IFNγ [42] and that IFNγ signalling is required for the induction of CXCR3 on CD4^+^ T cells [53]. Thus, the iron component of iron dextran is presumably responsible for the vast majority of changes measured in the FeD mice, especially given that the dextran component itself did not prevent the development of ECM.

Altogether, we have demonstrated that parenteral iron supplementation significantly decreases the incidence of ECM by reducing the accumulation of CD4^+^ and CD8^+^ T cells in the brain. The decreased sequestration was associated with attenuated CXCR3 expression and reduced IFNγ-responsiveness of splenic CD4^+^ T cells. To the best of our knowledge, this is the first study to report the potential of iron supplementation to prevent the development of ECM. We believe that a better understanding of the cellular and molecular mechanisms underlying the iron-mediated, immunomodulatory protection could aid in the development of new therapeutic strategies to treat malaria-infected individuals prior to the onset of cerebral malaria pathology.

## Materials and Methods

### Mice

Female, C57BL/6 mice (6–8 weeks old) were purchased from Charles River Laboratories and Jackson Laboratories, and were kept in pathogen-free housing. All research involving mice was carried out according to the regulations of the Canadian Council of Animal Care and was approved by the McGill University Animal Care Committee under ethics protocol number 5925. Mice were euthanized at established humane endpoints using CO_2_ asphyxiation followed by cervical dislocation or by using isoflurane if perfusion was performed.

### Parasites and infection

In all experiments, red blood cells infected with *Plasmodium berghei* ANKA parasites expressing a green fluorescent protein (GFP)-luciferase fusion protein were used (Malaria Research and Reference Reagent Resource Center). C57BL/6 mice were infected by intraperitoneal (i.p.) inoculation of 10^4^ infected red blood cells. In all experiments other than late-stage survival and survival and disease assessment, mice received either PBS or 4 mg of iron dextran for five days before and five days after parasite inoculation (infected groups) or mock infection (uninfected groups) by i.p. injection. For late-stage survival, mice received either PBS or 4mg of iron dextran for five days (or until they succumbed to ECM), starting on day 4 post-infection or day 5 post-infection. Unless otherwise stated, mice were sacrificed on day 7 post-infection or post-mock infection, upon the development of ECM symptoms in the infected control group.

### Survival and disease assessment

Mice received PBS, 4.4 mg of dextran (M_w_ = 5,000kDa or M_w_ = 70,000kDa), or 4 mg of iron dextran for five days before and five days after parasite inoculation by i.p. injection. Starting on day three post infection, tail-vein blood was collected daily. Blood smears were stained with Diff-Quik, and parasitemia was determined by counting at least 500 cells. Levels of parasitemia assessed using luciferase activity were determined using the Firefly Luciferase Assay Kit (Biotium, Inc.), according to the manufacturer’s protocol. Infected mice were monitored two to three times daily for clinical symptoms of ECM, including head deviation, hemi- or paraplegia, ataxia, convulsions and coma.

### Blood-brain barrier integrity

Upon the development of clinical symptoms in the infected control group, mice were i.p. injected with 0.3 mL of 2% Evans blue (Sigma-Aldrich). The mice were sacrificed 2 h thereafter, without perfusion, and brains and hearts were weighed and placed in formamide (Sigma-Aldrich) for 48 h at 37°C to extract the dye. Absorbance was measured at 620nm. The concentration of Evans blue in the brain was calculated using a standard curve prepared with known concentrations of Evans blue in formamide, and was normalized to the concentration in the heart.

### Luciferase assay

Mice were perfused with PBS and organs were frozen immediately in liquid nitrogen. Organs were homogenized in PBS containing 0.1mg/mL aprotinin (Roche) 0.05mg/mL leupeptin (Roche) and 1X Firefly lysis buffer using a PRO200 Hand-held Homogenizer (Harvard Apparatus Canada). After lysing on ice, the mixture was centrifuged at 13,000 rpm for 30 min. The luciferase activity of the supernatant was measured using the Firefly Luciferase Assay Kit (Biotium, Inc.), according to the manufacturer’s protocol.

### Cytokine multiplex assay

Levels of the cytokines IFNγ, TNFα, IL-10, IL-1β and IL-6 in serum samples were measured using a multiplex electrochemiluminescence assay (Meso Scale Discovery). Blood was collected from the saphenous vein. The serum samples were prepared and the assay was run according to the manufacturer’s protocol. The plate was read by an Imager 2400 plate reader (Meso Scale Discovery).

### Quantitative real-time PCR array analysis

Genes associated with the host immune response were quantitated with the Mouse Innate and Adaptive Immune Responses RT^2^ Profiler PCR Array (PAMM-052ZD, SABiosciences). Mice were perfused with PBS and organs were frozen immediately in liquid nitrogen. Total RNA was extracted from the spleen, liver and brain using TRIzol (Life Technologies), according to the manufacturer’s protocol. Extracted RNA was treated with RQ1 RNase-free DNase (Promega) and purified using the RNeasy Mini Kit (Qiagen). The RNA was then concentrated via phenol-chloroform extraction and reverse transcribed using RevertAid H Minus Reverse Transcriptase (Thermo Scientific) using random hexamers (Invitrogen). A standardized amount of cDNA was mixed with the RT^2^ SYBR Green qPCR Mastermix and added to the RT^2^ Profiler PCR Array. The array was run using the recommended conditions on a CFX96 Touch Real-Time PCR Detection System.

### Flow cytometric analysis of the brain

Flow cytometry was performed using a BD LSR Fortessa and results were analyzed using FlowJo version 9.6.2. Mice were perfused for the analysis of brain sequestered cells. Brains were digested in RPMI containing 1.6mg/mL collagenase (type IV; Sigma-Aldrich) and 200μg/mL DNase I (Sigma-Aldrich) at 37°C for 50 min. Cells were isolated using a Percoll gradient (GE Healthcare) and debris was filtered out using a 70μm nylon mesh. Cells were counted and labelled with LIVE/DEAD amine-reactive violet viability marker according to the manufacturer’s protocol (Invitrogen). The cells were blocked and labeled with FITC anti-CD45 (eBioscience; 30-F11), PE anti-CD11b (BD Pharmingen; M1/70), APC anti-CD4 (eBioscience; RM4-5) and PerCP-Cy5.5 anti-CD8 (eBioscience; 53–6.7).

### Flow cytometric analysis of the spleen

Flow cytometry was performed using a BD LSR Fortessa and a BD FACSCanto II and results were analyzed using FlowJo version 9.6.2. Mice were not perfused for the analysis of splenic cells. Splenocytes were isolated and erythrocytes were lysed in Tris-NH_4_Cl buffer. Cells were counted, blocked with and labelled with PerCP-Cy5.5 anti-CD4 (BD Pharmingen; RM4-5), APC-eFluor780 anti-CD8 (eBioscience; 53–6.7), APC anti-CXCR3 (BioLegend; CXCR3-173), FITC anti-CD62L (eBioscience; MEL-14), FITC anti-Foxp3 (eBioscience; FJK-16s), FITC anti-CD8 (eBioscience; 53–6.7), FITC anti-CD3 (eBioscience; 145-2C11), PE anti-CD25 (BD Pharmingen; PC61) PE anti-T-bet (eBioscience; eBio4B10), PE anti-NK1.1 (BD Pharmingen; PK136), PE-Cy7 anti-CD44 (BD Pharmingen; IM7), PE anti-CD11b (eBioscience; M1/70), PerCP-Cy5.5 anti-CD11c (eBioscience; N418), FITC anti-MHCII (eBioscience; M5/114.15.2), APC anti-CD40 (BD Pharmingen; 3/23), biotin rabbit anti-mouse CXCL10 (Cedarlane; BAF466), purified hamster anti-mouse IFNγR2 (BD Pharmingen; MOB-47), biotin mouse anti-Armenian and Syrian hamster (BD Pharmingen; 554010) and streptavidin APC-eFluor780 (eBioscience; 47-4317-82). Isolated splenocytes were labelled with CFSE and stimulated with 1μg/mL of anti-CD3 and 0.5μg/mL of anti-CD28. Cells were incubated at 37°C and collected on day 1 and 4 post stimulation. Cells were blocked and labelled with PerCP-Cy5.5 anti-CD4 (BD Pharmingen; RM4-5) and APC-eFluor780 anti-CD8 (eBioscience; 53–6.7).

### IFNγ Stimulation and Western Blot

Splenic CD4^+^ T cells were isolated using the Stemcell Technologies EasySep Mouse CD4^+^ T Cell Enrichment Kit, according to the manufacturer’s protocol. 1 x 10^6^ CD4^+^ T cells were stimulated with 1000U/mL of recombinant murine IFNγ (Invitrogen) for 0, 15, 30 and 60 minutes. Cells were lysed (50mM Tris (pH 7.0), 0.1 mM EDTA, 0.1 mM EGTA, 0.1% 2-mercaptoethanol, 1% IGEPAL, complete protease inhibitor (Roche), 1mM Na_3_VO_4_ and 50 mM NaF) and run on a SDS-PAGE gel. Proteins were detected using antibodies against pSTAT1 (Cell Signalling; D4A7) and STAT1 (Cell Signalling; 42H3). Anti-rabbit antibodies conjugated to horse-radish peroxidase (Amersham) were used as secondary antibodies. Membranes were visualised using the Pierce ECL Western Blotting Substrate (Thermo Fischer Scientific).

### Statistical analysis

Statistical analyses were performed using the unpaired Student’s *t*-test. Error bars represent S.E.M. The log-rank was used for all experiments in which survival was assessed as an endpoint. The data were analyzed using GraphPad Prism software (version 5.0). * *P* < 0.05, ** *P* < 0.01, and *** *P* < 0.001.

## Supporting Information

S1 File Figure A. **Iron Dextran Administered after Infection can Prevent the Development of ECM.** Survival of mice treated with PBS or iron dextran starting 4 or 5 days post-infection. The average of two individual experiments is shown. *n* = 17 for PBS mice; *n* = 9 for FeD mice, 4 days post-infection; and *n* = 8 for FeD mice, 5 days post-infection. PBS = control, FeD = iron dextran. Statistically significant differences, shown by asterisks (* *P* < 0.05), were determined by log-rank test. **Figure B. RLU Measured in the Blood, Brain, Spleen and Liver.** Parasitemia was determined by measuring relative luminescence units (RLU) per μL of blood in infected FeD mice and control mice (**a**). Parasite levels in the brain (**b**), spleen (**c**) and liver (**d**) on day 7 post-infection were determined by measuring RLU. For parasitemia: *n* = 10 for the control and FeD mice. For tissue parasite burden: *n* = 6 for all groups, except for the control, unsymptomatic mice (*n* = 4) and the control, symptomatic mice (*n* = 4). Shown on the graphs are the average ± S.E.M. FeD = iron dextran, PBS = control. Statistically significant differences, shown by asterisks (** *P* < 0.01 and *** *P* < 0.001), were determined by unpaired Student’s t-test. **Figure C. Systemic Inflammation in FeD Mice is Increased Only Late during the Infection.** Concentration of IFNγ (**a**), TNFα (**b**), IL-10 (**c**), IL-1β (**d**) and IL-6 (**e**) in the serum. *n* = 2 for all groups. FeD = iron dextran, PBS = control. **Table A. Immune Response-related Gene Expression in the Brain is Mostly Reduced or Unchanged in FeD Mice.** The mRNA expression of immune related genes was determined in the brain on day 7 post-infection. mRNA levels were normalized to *Gusb*. 2 samples pooled from 6 mice (3 mice per sample) were used for each group. Shown in the table are the averages UI = uninfected, I = infected, FeD = iron dextran, PBS = control. **Table B. Immune Response-related Gene Expression in the Spleen is Mostly Reduced or Unchanged in FeD Mice.** The mRNA expression of immune related genes was determined in the spleen on day 7 post-infection. mRNA levels were normalized to *Gusb*. 2 samples pooled from 6 mice (3 mice per sample) were used for each group. Shown in the table are the averages. UI = uninfected, I = infected, FeD = iron dextran, PBS = control. **Table C. Immune Response-related Gene Expression in the Liver is Mostly Reduced or Unchanged in FeD Mice**. The mRNA expression of immune related genes was determined in the liver on day 7 post-infection. mRNA levels were normalized to *Hsp90*. 2 samples pooled from 6 mice (3 mice per sample) were used for each group. Shown in the table are the averages. UI = uninfected, I = infected, FeD = iron dextran, PBS = control. **Figure D. Iron Dextran Causes a Minor Delay in the Proliferation of Splenic CD4** ^**+**^ **T Cells.** Representative flow cytometric histograms of CSFE after gating on CD4^+^ or CD8^+^ T cells on day 1 (**a**) and day 4 (**b**) after *ex vivo* stimulation. Splenic cells were isolated on day 7 post-infection. *n* = 6 for control mice and *n* = 7 for FeD mice. FeD = iron dextran, PBS = control. **Figure E. Percentage of Activated CD4** ^**+**^ **and CD8** ^**+**^ **T Cells in the Spleen is Slightly Decreased in the FeD Mice.** Representative flow cytometric dot plots and the percentage of CD62L^lo^CD25^+^ cells (**a**) and representative flow cytometric dot plots and the percentage of CD62L^lo^CD44^+^ cells (**b**) after gating on CD4^+^ or CD8^+^ T cells. All experiments were performed on day 7 post-infection. The numbers shown on the dot plots indicate the mean percentage of cells inside the gate ± S.E.M. Shown on the graphs are the average ± S.E.M *n* = 20 for all groups. The average of three individual experiments is shown. FeD = iron dextran, PBS = control. Statistically significant differences, shown by asterisks (*** *P* < 0.001), were determined by unpaired Student’s t-test. **Figure F. Iron Supplementation Results in a Slight Increase in the Priming Capacity of Splenic cDCs.** The percentage of CD11b^+^CD11c^+^ cells, and the MFI of CXCL10, CD40 and MHCII after gating on CD11b^+^CD11c^+^ cells on day 3 (**a**) and day 7 post-infection (**b**). On day 3 post-infection, *n* = 6 for control mice and *n* = 5 for FeD mice. On day 7 post-infection, *n* = 6 for control mice and *n* = 6 for FeD mice. Shown on the graphs are the average ± S.E.M FeD = iron dextran, PBS = control. Statistically significant differences, shown by asterisks (* *P* < 0.05 and ** *P* < 0.01), were determined by unpaired Student’s t-test. **Figure G. A Greater Percentage of Splenic CD4** ^**+**^ **T Cells in FeD Mice Show an Activated Phenotype and Express CXCR3 on Day 3.** Representative flow cytometric dot plots of CD62L^lo^CD25^+^ and CD62L^lo^CD44^+^ cells (**a**) and the percentage of CD62L^lo^CD25^+^ and CD62L^lo^CD44^+^ cells (**b**) after gating on CD4^+^ T cells. Representative flow cytometric dot plots of CXCR3^+^ CD4^+^ T cells (**c**), and the percentage of CXCR3^+^ cells and the MFI of CXCR3 (**d**) after gating on CD4^+^ T cells. All experiments were performed on day 3 post-infection. The numbers shown on the dot plots indicate the mean percentage of cells inside the gate ± S.E.M. Shown on the graphs are the average ± S.E.M *n* = 6 for control mice and *n* = 5 for FeD mice. FeD = iron dextran, PBS = control. Statistically significant differences, shown by asterisks (** *P* < 0.01 and *** *P* < 0.001), were determined by unpaired Student’s t-test. **Figure H. Attenuated Expression of IFNγR2 and T-bet does not Result in Decreased Expression of CXCR3 on Splenic CD8** ^**+**^ **T Cells.** Representative flow cytometric dot plots for IFNγR2^+^ CD8^+^ T cells (**a**) and the percentage of IFNγR2^+^ cells (**b**) after gating on CD8^+^ T cells on day 3 and day 7 post-infection. Representative flow cytometric dot plots for T-bet^+^ CD8^+^ T cells (**c**) and the percentage of T-bet^+^ cells (**d**) after gating on CD8^+^ T cells on day 3 and day 7 post-infection. The numbers shown on the dot plots indicate the mean percentage of cells inside the gate ± S.E.M. On day 3, *n* = 6 for control mice and *n* = 5 for FeD mice. On day 7, *n* = 6 for control mice and *n* = 6 for FeD mice. FeD = iron dextran, PBS = control. Statistically significant differences, shown by asterisks (** *P* < 0.01), were determined by unpaired Student’s t-test. **Figure I. Iron Supplementation Decreases STAT1 Phosphorylation in Splenic CD4** ^**+**^ **T cells in FeD Mice.** Representative western blot of pSTAT1 and STAT1. *n* = 2 for all groups. FeD = iron dextran, PBS = control.
(PDF)
