# Supplementary material for: Iron Prevents the Development of Experimental Cerebral Malaria by Attenuating CXCR3-Mediated T Cell Chemotaxis
Source: PLoS One. 2015 Mar 13;10(3):e0118451. doi: 10.1371/journal.pone.0118451 (PMC4359107; doi:10.1371/journal.pone.0118451)

Figure A

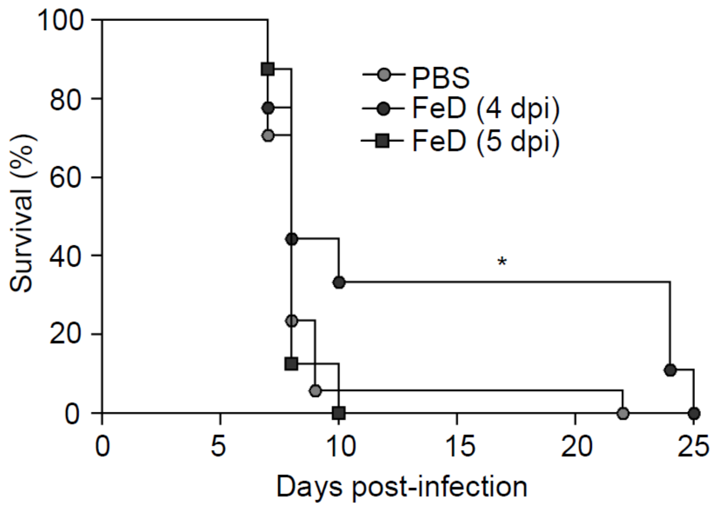

100
80
60
40
20
0
Survival (%)
0
5
10
15
20
25
Days post-infection
PBS
FeD (4 dpi)
FeD (5 dpi)
*

Figure B

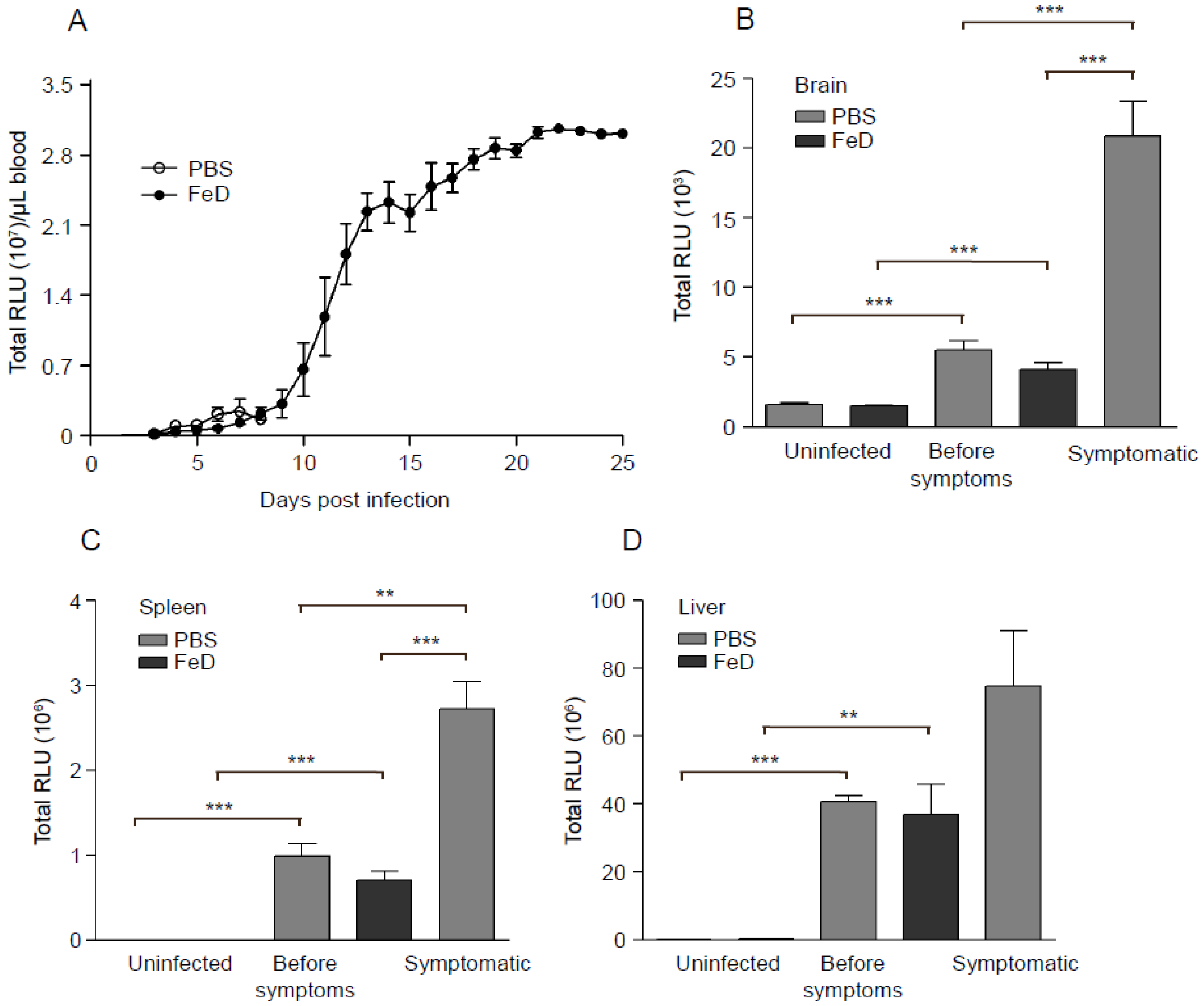
A
Total RLU (10⁷)/µL blood
3.5
2.8
2.1
1.4
0.7
0
PBS
FeD
0
5
10
15
20
25
Days post infection
B
Brain
PBS
FeD
Total RLU (10³)
25
20
15
10
5
0
***
***
***
***
Uninfected
Before symptoms
Symptomatic
C
Spleen
PBS
FeD
Total RLU (10⁶)
4
3
2
1
0
**
***
***
***
Uninfected
Before symptoms
Symptomatic
D
Liver
PBS
FeD
Total RLU (10⁶)
100
80
60
40
20
0
**
***
Uninfected
Before symptoms
Symptomatic

Figure C

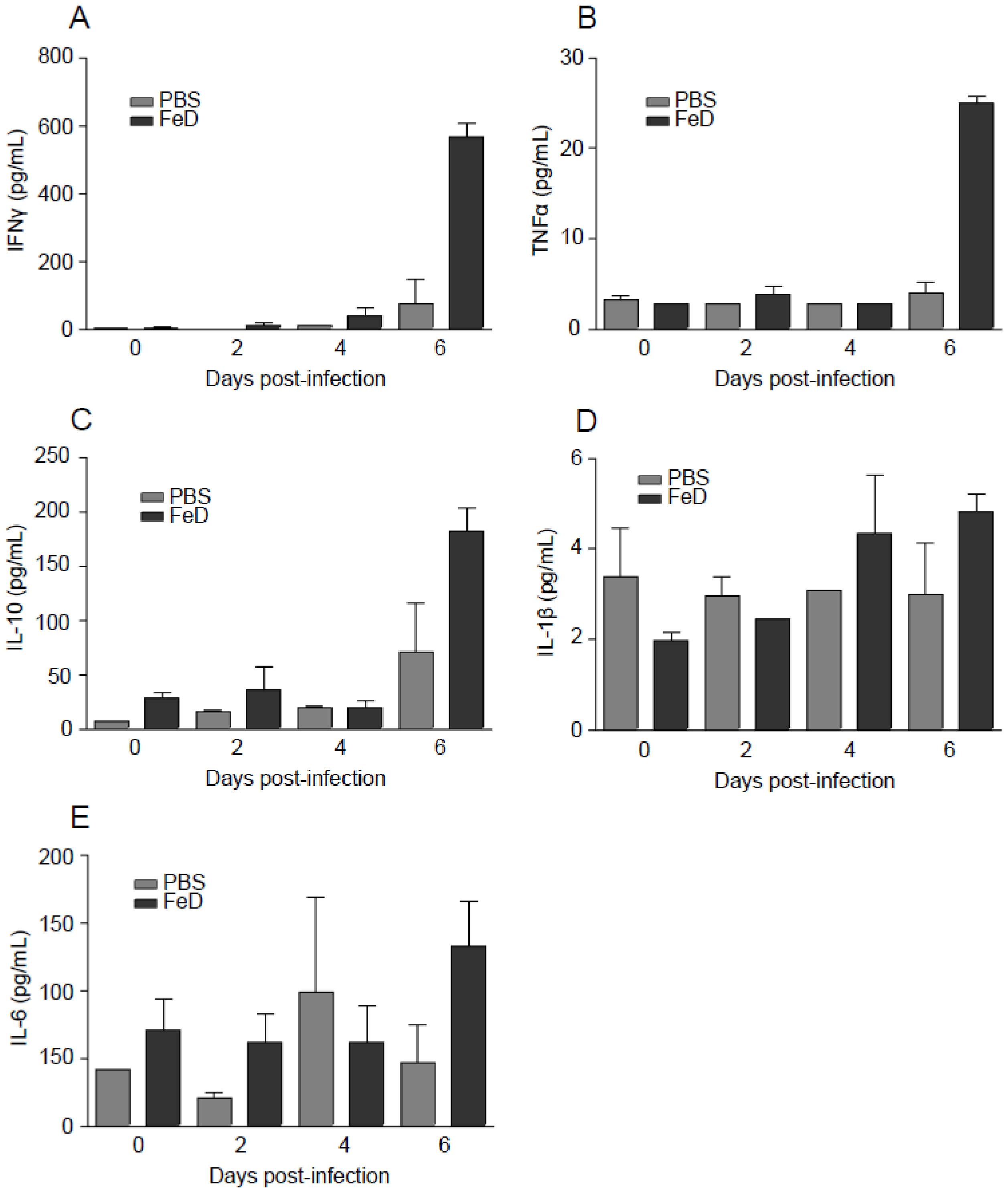
A
800
600
400
200
0
IFNγ (pg/mL)
PBS
FeD
0
2
4
6
Days post-infection
B
30
20
10
0
TNFα (pg/mL)
PBS
FeD
0
2
4
6
Days post-infection
C
250
200
150
100
50
0
IL-10 (pg/mL)
PBS
FeD
0
2
4
6
Days post-infection
D
6
4
2
0
IL-1β (pg/mL)
PBS
FeD
0
2
4
6
Days post-infection
E
200
150
100
150
0
IL-6 (pg/mL)
PBS
FeD
0
2
4
6
Days post-infection

Table A

gene	UI, PBS	UI, FeD	I, PBS	I, FeD	gene	UI, PBS	UI, FeD	I, PBS	I, FeD	gene	UI, PBS	UI, FeD	I, PBS	I, FeD
APCS	1.00	1.12	1.02	0.73	IFNA1	1.19	1.52	1.39	1.06	MX1	1.14	1.15	26.73	6.91
C3	1.47	0.40	3.63	2.02	IFNAR1	1.00	1.02	1.09	0.89	MYD88	1.04	1.20	3.75	2.29
C5R1	1.02	1.19	4.58	3.77	IFNB1	1.18	1.65	1.58	1.09	NKκB1	1.00	1.15	2.09	1.14
CASP1	1.00	1.01	3.95	1.63	IFNG	1.00	1.12	47.21	22.23	IκBKB	1.02	1.05	2.62	1.44
CCL12	1.05	1.31	20.81	30.46	IFNGR1	1.00	1.05	2.15	1.36	NLRP3	1.02	0.75	2.37	1.31
CCL5	1.01	1.06	18.20	7.15	IL-10	1.00	1.12	12.55	2.64	NOD1	1.04	1.17	2.76	2.16
CCR4	1.16	1.66	2.33	1.17	IL-13	1.02	1.50	1.67	1.10	NOD2	1.29	2.12	3.19	2.09
CCR5	1.03	1.20	4.23	2.01	IL-17A	1.01	1.11	1.60	1.10	RAG1	1.03	2.26	0.98	1.90
CCR6	1.10	1.73	0.92	1.35	IL-18	1.00	0.87	1.32	0.95	RORC	1.00	1.22	1.77	1.09
CCR8	1.00	1.12	1.06	0.73	IL-1α	1.04	0.84	2.88	1.71	SLC11A1	1.00	0.92	2.00	1.63
CD14	1.01	1.02	16.83	2.09	IL-1β	1.06	1.16	3.23	2.11	STAT1	1.00	0.94	12.71	7.54
CD4	1.00	0.68	1.12	0.80	IL-1R1	1.03	1.25	1.69	1.16	STAT3	1.00	1.01	3.28	1.71
CD40	1.01	1.00	1.73	1.71	IL-2	1.15	1.58	1.51	1.30	STAT4	1.03	0.77	1.77	0.96
CD40L	1.00	1.12	2.36	1.35	IL-23A	1.01	0.94	1.30	1.13	STAT6	1.00	1.14	1.67	1.23
CD80	1.00	0.61	4.82	1.37	IL-4	1.09	1.52	1.28	0.74	TBX21	1.00	1.12	2.91	1.11
CD86	1.00	0.79	4.71	1.95	IL-5	1.04	1.21	2.74	1.21	TICAM1	1.01	1.07	1.74	1.46
CD8α	1.13	1.37	34.56	17.01	IL-6	1.00	1.49	9.19	2.22	TLR1	1.00	0.82	3.76	2.80
CRP	1.49	0.43	0.54	1.07	IRAK1	1.00	1.04	1.03	0.76	TLR2	1.05	0.99	7.27	3.83
GM-CSF	1.24	1.33	2.51	1.35	IRF3	1.00	1.08	0.92	0.95	TLR3	1.00	1.08	3.14	1.73
CXCL10	1.01	1.01	308.53	166.16	IRF7	1.00	1.35	36.26	12.37	TLR4	1.00	0.87	2.16	1.72
CXCR3	1.05	1.16	9.84	3.81	ITGAM	1.00	0.72	1.17	0.99	TLR5	1.00	0.97	0.89	0.74
DDX58	1.00	1.00	8.71	4.36	JAK2	1.00	0.96	1.47	1.18	TLR6	1.03	0.93	1.85	1.35
FASL	1.00	1.12	1.34	0.73	LY96	1.00	0.44	1.34	0.98	TLR7	1.00	1.18	4.76	2.59
FOXP3	1.01	1.68	1.29	1.16	LYZ2	1.01	1.23	7.76	6.07	TLR8	1.15	1.65	3.91	2.36
GATA3	1.09	1.24	1.78	1.15	ERK2	1.00	0.91	1.18	0.79	TLR9	1.01	0.81	2.25	1.97
H2-Q10	1.25	0.49	0.83	0.61	JNK1	1.00	0.95	1.06	0.76	TNF	1.00	1.25	29.96	15.52
H2-T23	1.00	1.01	11.83	5.96	MBL2	1.39	1.85	1.28	1.17	TRAF6	1.02	1.06	1.67	1.24
ICAM1	1.08	1.58	14.34	9.25	MPO	1.00	0.81	1.11	0.74	TYK2	1.01	0.90	1.20	1.16

Table B

	UI, PBS	UI, FeD	I, PBS	I, FeD	gene	UI, PBS	UI, FeD	I, PBS	I, FeD	gene	UI, PBS	UI, FeD	I, PBS	I, FeD
APCS	1.00	4.41	2.01	1.44	IFNA1	1.01	4.97	7.28	7.49	MX1	1.01	0.42	4.96	1.79
C3	1.00	3.52	12.94	5.33	IFNAR1	1.00	3.57	7.40	3.97	MYD88	1.00	5.72	48.24	32.56
C5R1	1.02	24.57	327.10	285.60	IFNB1	1.00	5.19	5.42	5.49	NKκB1	1.00	2.06	2.39	2.51
CASP1	1.00	0.61	0.47	0.96	IFNG	1.09	1.35	58.21	72.15	IκBKB	1.00	0.45	1.09	0.77
CCL12	1.00	0.32	1.22	4.25	IFNGR1	1.00	1.53	5.30	2.78	NLRP3	1.00	2.95	16.39	13.07
CCL5	1.00	1.44	5.60	5.71	IL-10	1.06	9.22	144.40	164.27	NOD1	1.01	1.77	7.65	3.77
CCR4	1.00	3.09	3.14	2.49	IL-13	1.00	4.41	1.31	5.26	NOD2	1.01	6.53	5.71	5.08
CCR5	1.00	0.93	20.17	22.63	IL-17A	1.00	4.41	0.70	1.44	RAG1	1.00	4.41	0.70	1.44
CCR6	1.00	0.72	1.09	0.68	IL-18	1.00	1.16	6.43	2.42	RORC	1.00	5.63	1.85	1.60
CCR8	1.02	4.38	6.86	11.06	IL-1α	1.00	2.08	5.95	5.14	SLC11A1	1.00	9.38	31.46	31.09
CD14	1.00	0.76	2.85	1.31	IL-1β	1.00	1.39	0.90	0.88	STAT1	1.00	1.36	3.47	4.48
CD4	1.00	8.87	18.14	8.19	IL-1R1	1.00	5.82	9.47	5.18	STAT3	1.00	3.86	10.48	7.10
CD40	1.00	0.69	0.42	0.50	IL-2	1.00	2.74	5.03	2.03	STAT4	1.01	2.40	3.81	4.33
CD40L	1.00	7.95	8.62	19.63	IL-23A	1.00	4.41	5.13	3.06	STAT6	1.00	5.32	12.27	7.69
CD80	1.00	1.38	4.02	1.46	IL-4	1.00	0.56	0.38	0.54	TBX21	1.00	7.33	32.61	65.27
CD86	1.00	1.83	2.25	2.07	IL-5	1.00	4.41	11.05	2.71	TICAM1	1.02	9.23	38.66	17.39
CD8α	1.00	7.70	6.98	11.14	IL-6	1.00	4.41	7.33	5.52	TLR1	1.00	0.82	0.89	0.75
CRP	1.00	4.41	1.06	2.57	IRAK1	1.00	1.01	3.34	1.55	TLR2	1.00	2.56	5.16	3.03
GM-CSF	1.00	19.67	11.01	34.70	IRF3	1.00	1.25	0.81	1.12	TLR3	1.00	0.57	1.54	0.95
CXCL10	1.04	7.76	33.25	75.46	IRF7	1.01	7.02	37.11	41.92	TLR4	1.00	2.48	10.79	6.84
CXCR3	1.00	1.86	37.33	17.77	ITGAM	1.00	17.05	19.99	28.03	TLR5	1.01	1.37	1.87	0.56
DDX58	1.00	1.48	3.18	1.99	JAK2	1.00	1.12	1.62	1.22	TLR6	1.01	1.57	11.51	4.82
FASL	1.00	0.17	0.40	0.27	LY96	1.00	2.42	6.22	3.26	TLR7	1.00	1.19	11.33	3.34
FOXP3	1.00	7.46	6.51	2.54	LYZ2	1.00	0.93	1.57	1.17	TLR8	1.07	1.90	31.28	7.65
GATA3	1.01	3.82	3.37	7.53	ERK2	1.00	2.31	3.44	2.62	TLR9	1.00	9.59	16.23	12.36
H2-Q10	1.12	2.61	2.81	3.04	JNK1	1.00	3.62	3.47	2.62	TNF	1.00	8.79	20.32	13.16
H2-T23	1.00	0.59	0.74	0.89	MBL2	1.00	4.41	0.86	1.44	TRAF6	1.00	1.88	15.28	3.07
ICAM1	1.00	2.53	6.88	5.96	MPO	1.32	5.08	15.66	9.75	TYK2	1.00	5.30	4.73	4.37

Table C

gene	UI, PBS	UI, FeD	I, PBS	I, FeD	gene	UI, PBS	UI, FeD	I, PBS	I, FeD	gene	UI, PBS	UI, FeD	I, PBS	I, FeD
APCS	1.00	0.32	2.63	1.05	IFNA1	1.00	0.76	1.42	0.22	MX1	1.06	0.70	5.59	2.52
C3	1.05	0.56	1.24	0.51	IFNAR1	1.00	0.91	1.27	0.58	MYD88	1.03	0.69	3.85	1.58
C5R1	1.06	1.41	8.08	2.85	IFNB1	1.00	0.50	1.40	0.14	NKκB1	1.00	0.89	1.34	1.19
CASP1	1.00	1.83	6.80	9.79	IFNG	1.00	1.63	110.15	77.47	IκBKB	1.04	0.79	1.56	0.99
CCL12	1.01	1.52	11.30	31.70	IFNGR1	1.00	0.97	2.66	1.27	NLRP3	1.00	1.04	8.34	4.08
CCL5	1.02	1.42	22.37	18.45	IL-10	1.01	0.32	35.11	12.36	NOD1	1.00	1.01	3.06	1.58
CCR4	1.02	1.70	2.41	1.73	IL-13	1.01	1.26	1.38	1.03	NOD2	1.00	0.68	1.99	1.43
CCR5	1.00	0.20	8.57	3.88	IL-17A	1.01	1.26	1.38	1.03	RAG1	1.01	1.26	1.38	1.03
CCR6	1.02	0.39	0.91	0.28	IL-18	1.00	0.90	1.50	0.67	RORC	1.01	0.91	2.72	0.20
CCR8	1.01	1.26	5.83	4.21	IL-1α	1.00	0.90	1.21	0.91	SLC11A1	1.01	3.70	9.40	8.86
CD14	1.00	0.35	2.12	1.63	IL-1β	1.04	0.28	0.39	0.61	STAT1	1.00	1.05	5.23	6.57
CD4	1.04	3.68	9.88	5.27	IL-1R1	1.07	0.57	5.04	1.08	STAT3	1.00	0.89	1.94	1.11
CD40	1.01	1.15	4.77	6.96	IL-2	1.00	1.00	2.12	0.82	STAT4	1.00	0.87	9.77	7.55
CD40L	1.00	1.55	19.51	13.53	IL-23A	1.01	0.89	1.58	0.45	STAT6	1.02	0.72	1.22	0.78
CD80	1.00	1.92	8.79	3.35	IL-4	1.00	0.56	1.33	0.30	TBX21	1.00	1.23	10.39	9.69
CD86	1.00	0.82	3.96	2.36	IL-5	1.00	0.59	2.35	0.38	TICAM1	1.03	0.74	1.41	0.39
CD8α	1.00	1.45	32.42	26.48	IL-6	3.41	0.12	0.16	0.14	TLR1	1.01	2.51	10.83	6.20
CRP	1.00	0.33	0.67	0.29	IRAK1	1.00	1.24	1.38	0.51	TLR2	1.00	1.03	3.47	6.49
GM-CSF	1.10	0.86	1.25	0.40	IRF3	1.00	0.88	0.76	0.64	TLR3	1.01	0.66	2.01	1.15
CXCL10	1.00	2.99	10.36	18.44	IRF7	1.00	1.67	5.94	3.31	TLR4	1.00	1.72	5.91	3.16
CXCR3	1.01	0.85	11.11	5.54	ITGAM	1.00	1.37	16.35	8.27	TLR5	1.01	0.30	4.04	0.12
DDX58	1.00	0.90	1.13	0.80	JAK2	1.01	1.03	2.27	1.45	TLR6	1.00	1.03	4.72	2.15
FASL	1.01	1.26	4.02	1.65	LY96	1.00	0.64	1.45	0.65	TLR7	1.02	1.17	5.57	1.37
FOXP3	1.00	1.40	2.71	1.18	LYZ2	1.01	3.18	1.35	2.51	TLR8	1.01	2.04	8.94	2.32
GATA3	1.00	2.35	2.98	2.63	ERK2	1.00	0.72	1.09	0.59	TLR9	1.00	2.14	12.89	8.52
H2-Q10	1.00	0.84	0.54	0.26	JNK1	1.00	0.68	1.86	0.51	TNF	1.00	2.39	11.39	10.74
H2-T23	1.00	0.94	3.69	2.43	MBL2	1.01	0.51	0.33	0.32	TRAF6	1.00	1.09	3.17	0.56
ICAM1	1.00	1.69	5.24	4.99	MPO	1.01	1.40	2.38	2.17	TYK2	1.00	0.92	1.17	0.88

Figure D

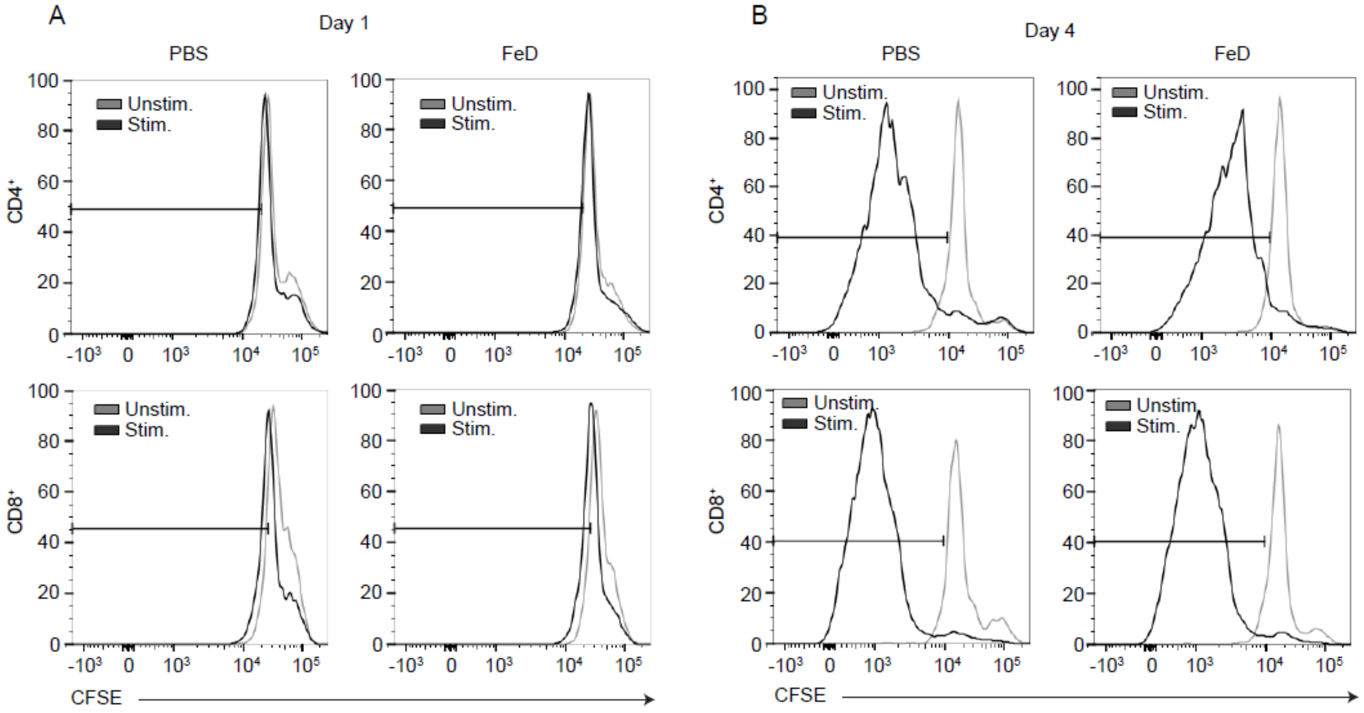

Figure E

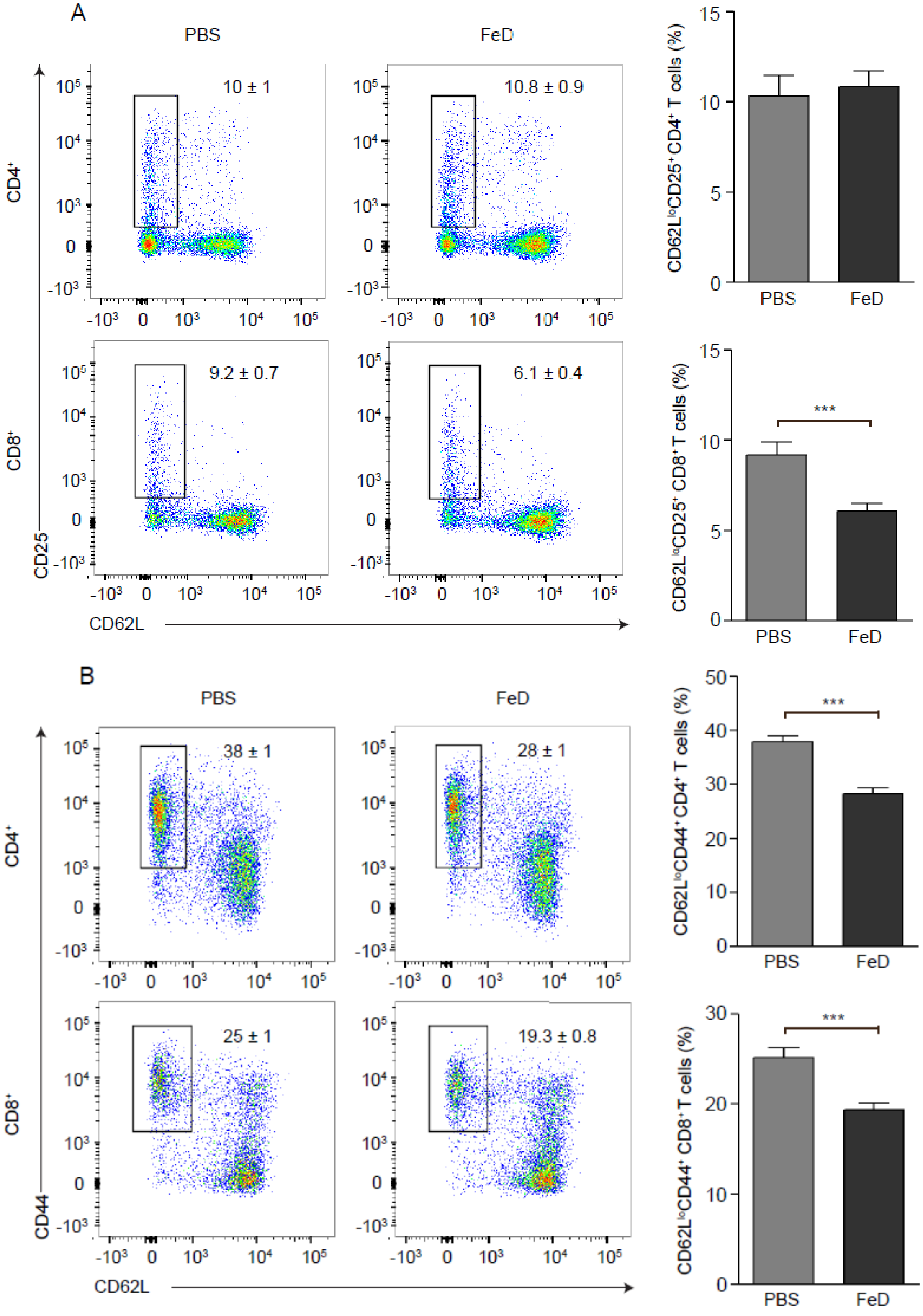
A
PBS
FeD
CD4+
CD8+
CD25
CD62L
10 ± 1
10.8 ± 0.9
9.2 ± 0.7
6.1 ± 0.4
CD62L^loCD25+CD4+ T cells (%)
CD62L^loCD25+ CD8+ T cells (%)
***
B
CD44
38 ± 1
28 ± 1
25 ± 1
19.3 ± 0.8
CD62L^loCD44+CD4+ T cells (%)
CD62L^loCD44+ CD8+ T cells (%)

Figure F

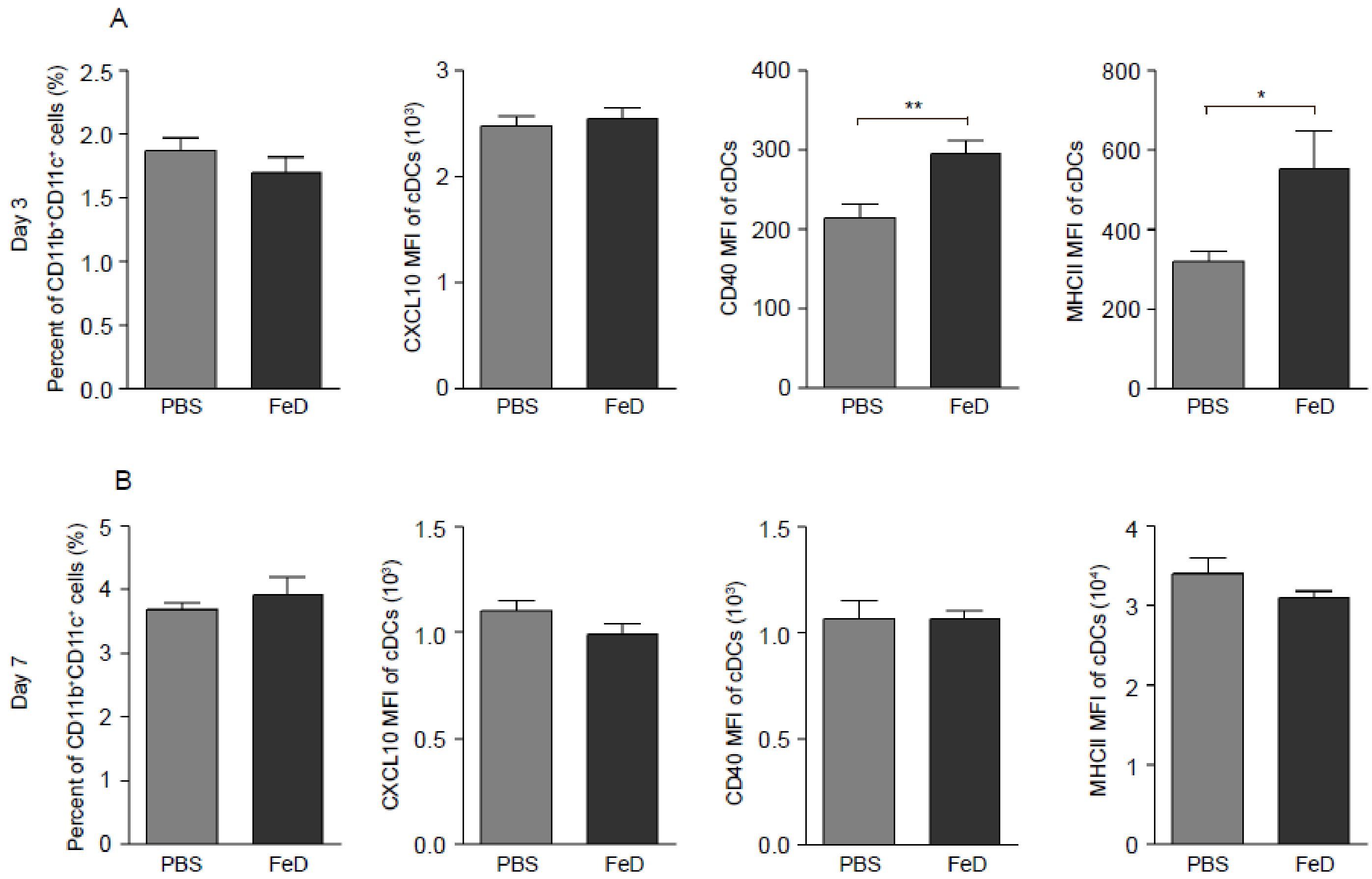
A
B
Day 3
Day 7
Percent of CD11b⁺CD11c⁺ cells (%)
CXCL10 MFI of cDCs (10³)
CD40 MFI of cDCs
MHCII MFI of cDCs
CD40 MFI of cDCs (10³)
MHCII MFI of cDCs (10⁴)
**
*
PBS
FeD

Figure G

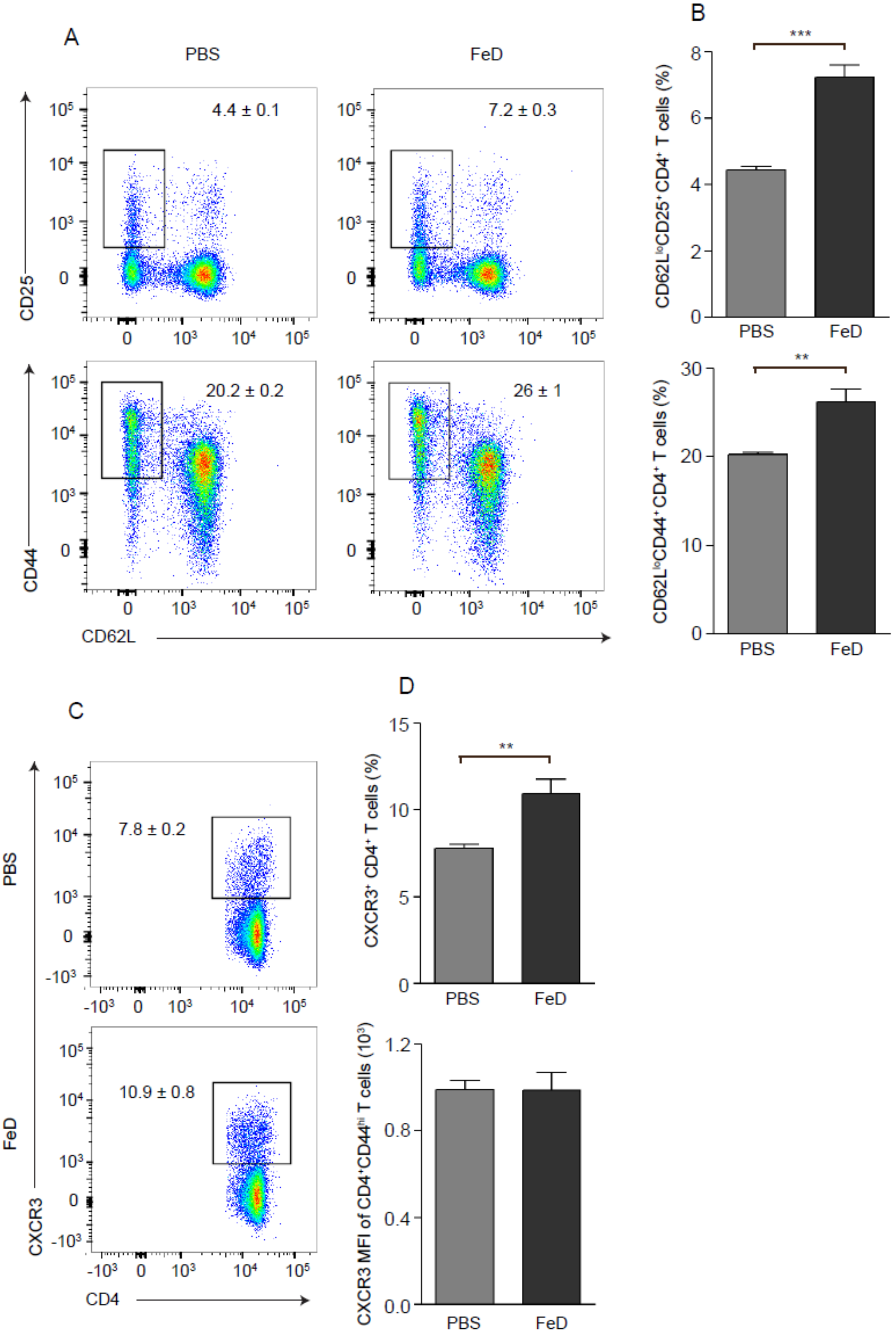

A
PBS
FeD
4.4 ± 0.1
7.2 ± 0.3
20.2 ± 0.2
26 ± 1
CD25
CD44
CD62L
B
CD62L^lo CD25^+ CD4^+ T cells (%)
CD62L^lo CD44^+ CD4^+ T cells (%)
***
**
C
7.8 ± 0.2
10.9 ± 0.8
CXCR3
CD4
D
CXCR3^+ CD4^+ T cells (%)
CXCR3 MFI of CD4^+ CD44^hi T cells (10^3)

Figure H

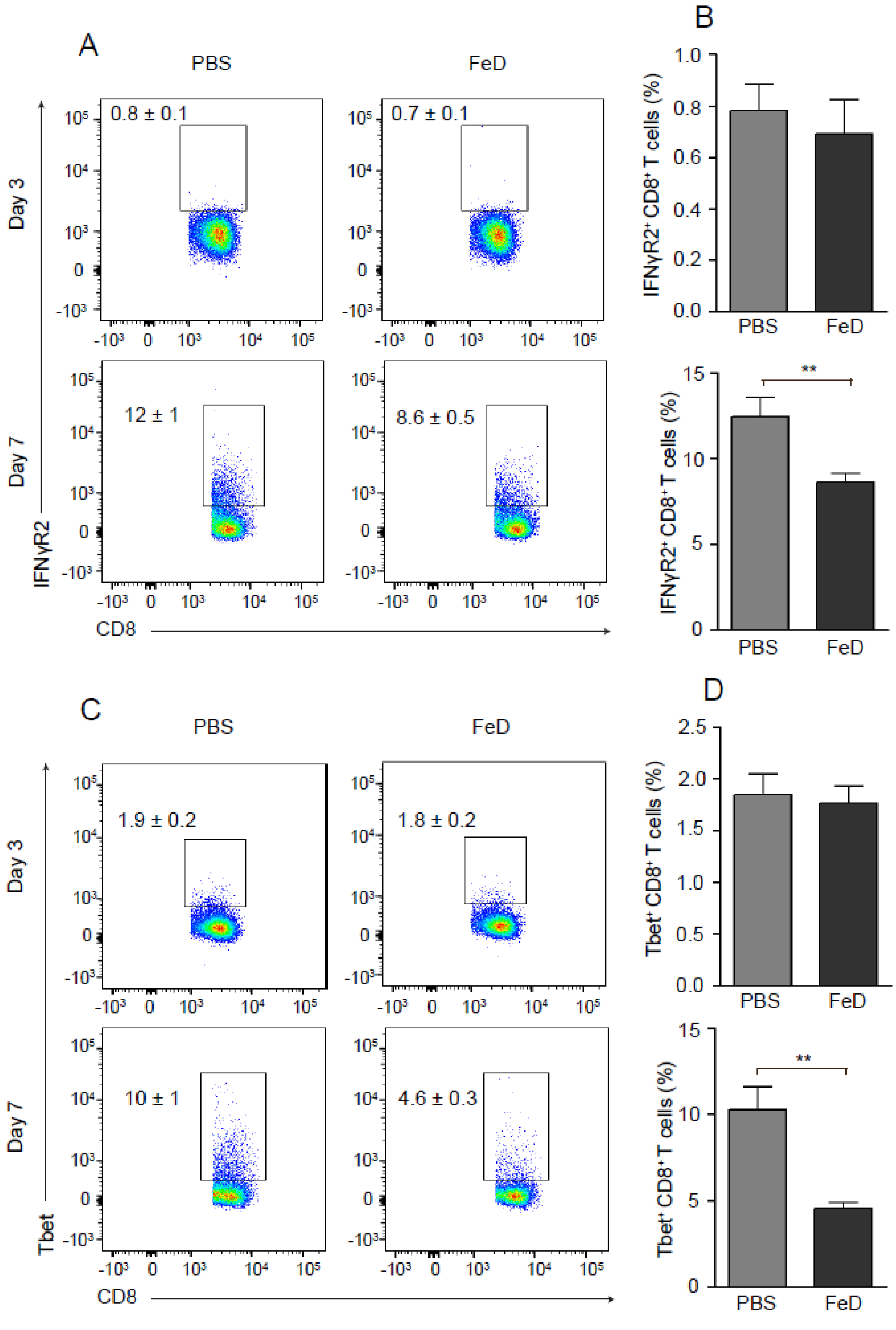
A
PBS
FeD
Day 3
Day 7
IFNγR2
CD8
0.8 ± 0.1
0.7 ± 0.1
12 ± 1
8.6 ± 0.5
B
IFNγR2+ CD8+ T cells (%)
PBS
FeD
**
C
PBS
FeD
Day 3
Day 7
Tbet
CD8
1.9 ± 0.2
1.8 ± 0.2
10 ± 1
4.6 ± 0.3
D
Tbet+ CD8+ T cells (%)
PBS
FeD
**

Figure I

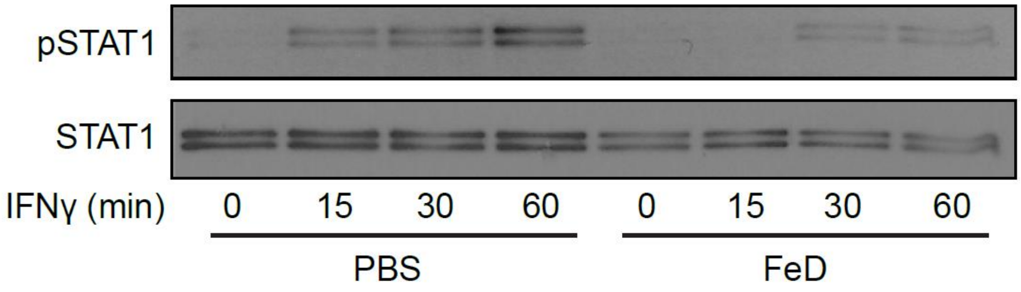

Supplement: S1 File — Iron Dextran Administered after Infection can Prevent the Development of ECM. Survival of mice treated with PBS or iron dextran starting 4 or 5 days post-infection. The average of two individual experiments is shown. n = 17 for PBS mice; n = 9 for FeD mice, 4 days post-infection; and n = 8 for FeD mice, 5 days post-infection. PBS = control, FeD = iron dextran. Statistically significant differences, shown by asterisks (* P < 0.05), were determined by log-rank test. Figure B. RLU Measured in the Blood, Brain, Spleen and Liver. Parasitemia was determined by measuring relative luminescence units (RLU) per μL of blood in infected FeD mice and control mice (a). Parasite levels in the brain (b), spleen (c) and liver (d) on day 7 post-infection were determined by measuring RLU. For parasitemia: n = 10 for the control and FeD mice. For tissue parasite burden: n = 6 for all groups, except for the control, unsymptomatic mice (n = 4) and the control, symptomatic mice (n = 4). Shown on the graphs are the average ± S.E.M. FeD = iron dextran, PBS = control. Statistically significant differences, shown by asterisks (** P < 0.01 and *** P < 0.001), were determined by unpaired Student’s t-test. Figure C. Systemic Inflammation in FeD Mice is Increased Only Late during the Infection. Concentration of IFNγ (a), TNFα (b), IL-10 (c), IL-1β (d) and IL-6 (e) in the serum. n = 2 for all groups. FeD = iron dextran, PBS = control. Table A. Immune Response-related Gene Expression in the Brain is Mostly Reduced or Unchanged in FeD Mice. The mRNA expression of immune related genes was determined in the brain on day 7 post-infection. mRNA levels were normalized to Gusb. 2 samples pooled from 6 mice (3 mice per sample) were used for each group. Shown in the table are the averages UI = uninfected, I = infected, FeD = iron dextran, PBS = control. Table B. Immune Response-related Gene Expression in the Spleen is Mostly Reduced or Unchanged in FeD Mice. The mRNA expression of immune related genes was d [file pone.0118451.s001.pdf]
